# TstI, a Type II restriction–modification protein with DNA recognition, cleavage and methylation functions in a single polypeptide

**DOI:** 10.1093/nar/gku187

**Published:** 2014-03-14

**Authors:** Rachel M. Smith, Christian Pernstich, Stephen E. Halford

**Affiliations:** The DNA-proteins Interaction Unit, School of Biochemistry, University of Bristol, University Walk, Bristol BS8 1TD, UK

## Abstract

Type II restriction–modification systems cleave and methylate DNA at specific sequences. However, the Type IIB systems look more like Type I than conventional Type II schemes as they employ the same protein for both restriction and modification and for DNA recognition. Several Type IIB proteins, including the archetype BcgI, are assemblies of two polypeptides: one with endonuclease and methyltransferase roles, another for DNA recognition. Conversely, some IIB proteins express all three functions from separate segments of a single polypeptide. This study analysed one such single-chain protein, TstI. Comparison with BcgI showed that the one- and the two-polypeptide systems differ markedly. Unlike the heterologous assembly of BcgI, TstI forms a homotetramer. The tetramer bridges two recognition sites before eventually cutting the DNA in both strands on both sides of the sites, but at each site the first double-strand break is made long before the second. In contrast, BcgI cuts all eight target bonds at two sites in a single step. TstI also differs from BcgI in either methylating or cleaving unmodified sites at similar rates. The site may thus be modified before TstI can make the second double-strand break. TstI MTase acts best at hemi-methylated sites.

## INTRODUCTION

Prokaryotes possess extensive arsenals of weapons to defend against bacteriophage (1). The most prevalent, and thus perhaps the most crucial, are the restriction–modification (RM) systems. Of the several thousand bacterial and archaeal genomes sequenced to date, only 5% lack candidate genes for an RM system while many possess multiple RM schemes, often >10 (2). Other means of defence against bacteriophage are less widespread across prokaryotic genera: for example, CRISPR loci, though found in ∼90% of archaea, are present in only 50% of bacteria (3). RM systems defend against phage by using two enzyme activities to distinguish between self and foreign DNA and to destroy the latter (4,5). One is a modification methyltransferase (MTase), which transfers a methyl group from S-adenosylmethionine (SAM) to an adenine or a cytosine within a particular DNA sequence, the recognition site for that system (6). The second is the restriction endonuclease (REase), which cleaves DNA with unmethylated (UM) recognition sites. It cannot cleave fully methylated (FM) DNA modified in both strands, nor hemi-methylated (HM) sites modified in one strand. Self DNA is thus never cleaved by the REase since semi-conservative replication of DNA previously modified in both strands yields the HM form. On the other hand, foreign DNA that lacks the appropriate methylation is destroyed as it enters the cell unless the MTase protects every recognition site before the REase acts at any one site (7).

Most RM systems fall into either Type I or Type II categories (5,8). Type I schemes usually feature a single protein comprised of separate subunits for DNA cleavage (R), methylation (M) and sequence specificity (S) in an R_2_M_2_S assembly (7,9,10). The R subunit possesses an ATP-dependent DNA translocase so that even though DNA cleavage is elicited by an UM site, it can occur kb away from the site. In some cases, the R, M and S functions are all carried as domains within a single polypeptide, which also has the translocase activity associated with R (11,12). In contrast, Type II RM systems generally feature two separate proteins, for restriction and modification respectively (6). The REase is typically a homodimer that cuts both DNA strands at fixed loci within a palindromic sequence (13,14). The MTase, often a monomer, acts at the same sequence as the REase, ideally its HM state to which it transfers one methyl group to a base in the unmodified strand (15,16). However, many Type II systems deviate from the orthodox in at least one of the following respects: gene organisation; reaction mechanism; nature of recognition sequence; DNA cleavage loci. The unorthodox systems can be classified on the basis of these factors into several sub-types: IIA, IIB, IIC and so forth (8). The only feature common to all Type II REases is that, unlike Type I, they cleave DNA at fixed positions relative to their recognition sites.

The Type IIB subset perhaps differ the most from the orthodox (17). Firstly, instead of separate proteins for restriction and modification, the IIB systems employ the same protein for both activities. In many cases, including the archetype of this subset, BcgI, the protein contains two different subunits, A and B, often (but not always; 18) in a 2:1 ratio (19–22). The A chain possesses the amino acid sequences for both endonuclease and MTase active sites while the B chain resembles a Type I S subunit and likewise identifies the recognition sequence (20,23). The A_2_B assembly of BcgI is thus analogous to the R_2_M_2_S arrangement of a Type I protein, with BcgIA reflecting a fusion of R and M subunits albeit without the translocase function. However, just as some Type I systems carry all three functions in a single polypeptide (11,12), several Type IIB systems also comprise a single polypeptide chain: these include HaeIV (24), AloI (25) and others related to AloI such as TstI (26). The HaeIV and the AloI polypeptides appear to associate in solution to homodimers or homotetramers, respectively.

Second, nearly all Type IIB proteins recognise bipartite non-palindromic sequences consisting of two specified segments, each 2–4 bp long, separated by 5–7 bp of undefined sequence (2,17). Sites of this sort are characteristic of Type I rather than Type II systems (9,10,23,26). The restriction function of the IIB protein then cuts both strands of the DNA at fixed distances away from the site, on both sides of the site, while the modification function transfers methyl groups from SAM to two specified adenines, one to each component of the bipartite sequence in opposite strands. For example, the recognition sequence for the two-chain system BcgI (19) is

\begin{eqnarray*} &&5' - \downarrow ({\rm N})_{10} - {\rm CG}\underline A - ({\rm N})_6 - {\rm TGC} - ({\rm N})_{12} \downarrow - 3'\\ &&3' - \uparrow ({\rm N})_{12} - {\rm GCT} - ({\rm N})_6 - \underline A{\rm CG} - ({\rm N})_{10} \uparrow - 5' \end{eqnarray*}

while that for the single-chain protein TstI (2) is

\begin{eqnarray*} &&5' - \downarrow ({\rm N})_8 - {\rm C}\underline A {\rm C} - ({\rm N})_6 - {\rm TCC} - ({\rm N})_{12} \downarrow - 3'\\ &&3' - \uparrow ({\rm N})_{13} - {\rm GTG} - ({\rm N})_6 - \underline A{\rm GG} - ({\rm N})_7 \uparrow - 5' \end{eqnarray*}

[N is any nucleotide; *A* underlined in italics the methylation sites; arrows the cleavage loci.] The REases thus excise a short fragment carrying the recognition site from the remainder of the DNA: in the case of TstI, a 27 bp duplex with 5 nt extensions at both 3′ ends. This fragment will be called the 32-mer as both strands are 32 nt long. Hence, the third distinctive feature of the IIB Type REases is that they make two rather than one double-strand break (DSB) at each site.

Fourth, while the orthodox Type II REases bind to solitary recognition sites and cleave the DNA one site at a time (13,14), nearly all of the Type IIB RM proteins, including BcgI (27), need to interact with two sites before the nuclease can cut the DNA (28). The conventional dimeric REases bind symmetrically to their palindromic recognition sequences, with each subunit contacting one half of the site. The active site from each subunit attacks the scissile bond in one strand, so the dimer makes one DSB at a single site. The requirement for two recognition sites is not unique to the IIB subset since this is also a feature of several other subsets (29–32). Such enzymes generally cleave DNA with two recognition sites *in cis* more readily than DNA with one site but they can still cleave one-site DNA, albeit inefficiently: either by a residual activity when bound to a solitary site or by interacting *in trans* with two separate DNA molecules (33–36). The BcgI REase follows the latter route as it has no activity when bound to one site (37). Its ability to act *in trans* was demonstrated by finding that its reaction on a plasmid with one BcgI site was enhanced by adding an oligoduplex carrying the cognate sequence (37), as is often the case with enzymes needing two sites (32). In contrast, the MTase activity of the BcgI protein did not need a second copy of the recognition sequence and was fully functional at a single HM site (38).

The Type II enzymes that require two sites generate various outcomes (30–35): some cleave the four target phosphodiester bonds (two at each site) in separate reactions, one bond at a time; others make a DSB at one of the two sites, the other site being used as an activator rather than as a substrate; further enzymes cleave all four scissile bonds in a concerted process, without liberating intermediates cut at some but not all bonds. Concerted action at two sites by a Type IIB REase requires parallel reactions at eight phosphodiester bonds, four at each site. Nevertheless, the BcgI REase cleaved a plasmid with two BcgI sites directly to the final product cut in both strands on both sides of both sites, all within the lifetime of a single DNA–protein complex (37). In order to cut the eight scissile bonds at the two target sites, the complex for DNA cleavage by BcgI probably needs four copies of the A_2_B protomer, bridging two copies of the recognition sequence, to give eight catalytic centres for phosphodiester hydrolysis, one in each A subunit (22). The MTase activity of BcgI also needed multiple copies of the A_2_B protein even though each A subunit possesses the catalytic functions for the transfer of one methyl group to a HM site (38).

Finally, some but not all Type IIB RM proteins have atypical co-factor requirements (17,40). They all need Mg^2+^ ions for their REase and SAM for their MTase activities, as is usual for REases and MTases respectively (13–16). However, for some Type IIB systems and likewise some other subsets (8), the REase requires not only Mg^2+^ but also the MTase co-factor SAM. The BcgI REase is one example, as are most of the two-chain IIB proteins (18–21): BcgI has no cutting activity in the absence of SAM (19,40). In contrast, the majority of the single-chain Type IIB proteins do not need SAM for their REase role: viz. HaeIV, AloI and TstI (24–26). In the presence of both SAM and Mg^2+^, as will be the case *in vivo*, a protein with both REase and MTase activities has the potential to either cleave or methylate an UM recognition site. If the two processes are similarly efficient, the system might be relatively incompetent at restricting foreign DNA as the UM DNA could be modified rather than restricted. But many proteins with both activities in the same assembly operate at UM sites solely as REases and not as MTases, so these still restrict foreign DNA (7,9,10): for example, the MTase component of BcgI is only active at HM sites (38) so UM sites are invariably cleaved rather than methylated.

Most of the information currently available about Type IIB RM systems relates to the two-chain protein BcgI (19,20,22,23,27,28,37,40). None of the single-chain IIB proteins have been examined in as much depth as BcgI. The objective of this study was to characterise the organisation, and both the REase and MTase activities, of a single-chain Type IIB system, in order to reveal the similarities and/or differences between this system and the well-characterised two-chain protein BcgI. The system chosen was TstI, since it is the only such protein to originate from a thermophilic organism, *Thermus scotoductus* (2), a species that grows at 70°C. Proteins from thermophiles are often more amenable to study than equivalent proteins from mesophiles.

## MATERIALS AND METHODS

### TstI protein

The 3756 bp *tsti* gene from *T. scotoductus* RFL1 (GI:149391961, AM410095.1) was reconfigured using the Life Technologies GeneOptimizer tool with, wherever possible, optimal codons for expression in *Escherichia coli* and with various novel restriction sites at the termini and at several internal loci: the reconfiguration retained the amino acid sequence of the TstI RM protein (Supplementary Figure S1). A 3779 bp segment of duplex DNA encompassing this sequence was synthesised by Life Technologies. The section of the synthesised DNA containing the *tsti* gene was excised using NdeI and BamHI and the fragment ligated to the NdeI and BamHI sites of pET15b and pET21a (Novagen) to create pET15b-TstI and pET21a-TstI respectively. The former expresses an N-terminal His tagged form of the TstI protein containing 1271 amino acids and the latter the native protein of 1251 amino acids. The expression plasmids were validated by sequencing (Eurofins MWG Operon). The plasmids were used separately to transform *E. coli* T7 Express *lysY/I^q^* (NEB). The His-tagged and the native proteins were purified from the cells with pET15b-TstI and pET21a-TstI, respectively, as described in Supplementary Data. Concentrations of pure TstI protein, either His-tagged or native, were evaluated from *A*_280_ readings using a molar extinction coefficient of 142,295 M^−1^ cm^−1^ for the polypeptide (calculated in ProtParam). Molar concentrations of TstI are given in terms of the tetrameric protein.

### DNA

**Figure 1.**
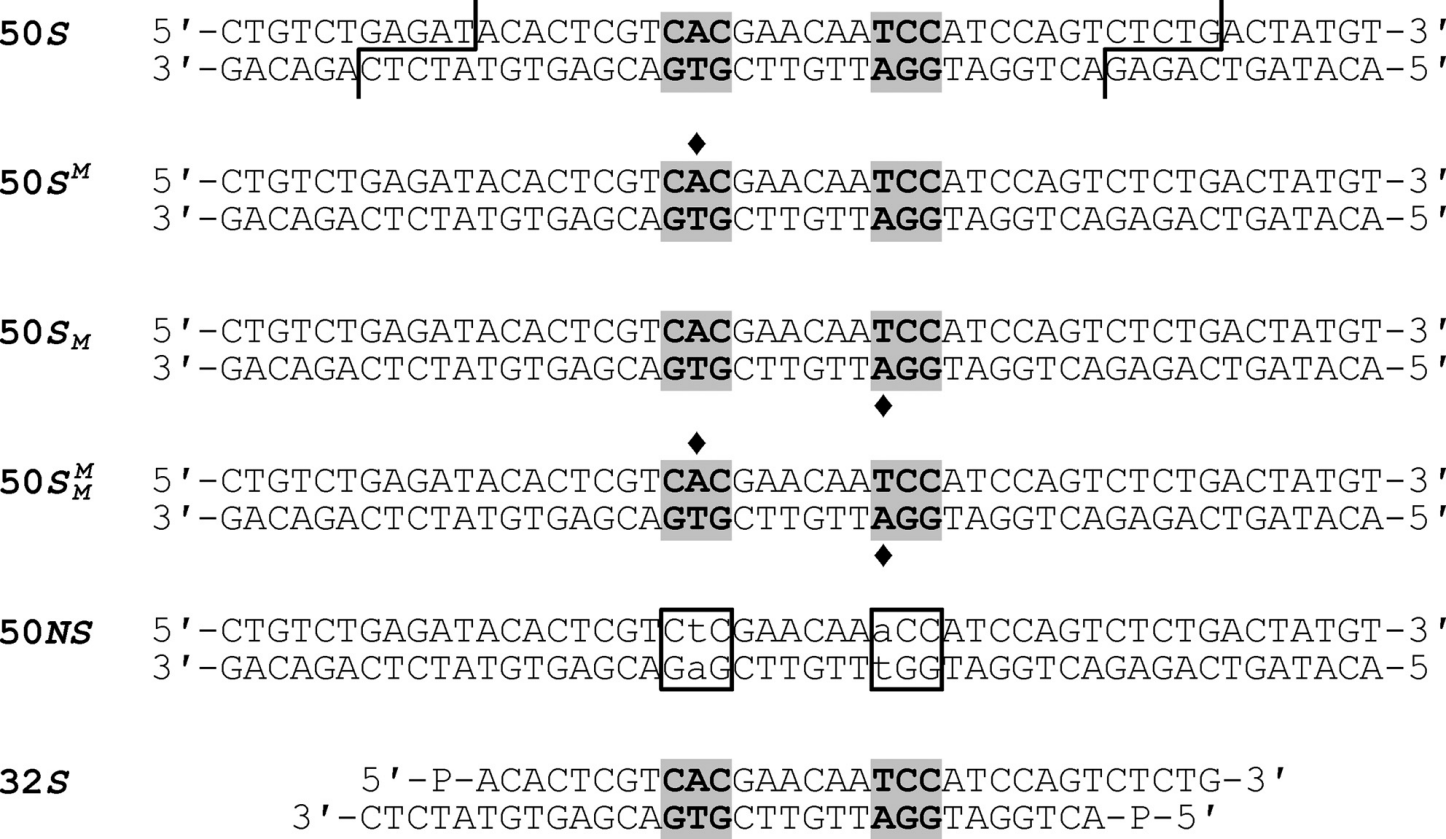
Oligoduplexes. The oligoduplexes used in this study are named from their lengths in nt and have the sequences indicated. In duplexes marked *S* (for specific), the bipartite recognition sequence for TstI is shaded in grey. The positions of DNA cleavage by the TstI REase are indicated in the 50*S* oligoduplex. The diamonds in 50*S^M^*, 50*S_M_* and $50S_M^M$ mark the location(s) where the duplex in question carries m6A in place of adenine to mimic the sites of methylation by the TstI MTase. In the non-specific duplex 50*NS*, the recognition sequence was disrupted (outlined boxes) by changing the two adenine residues modified by the TstI MTase to thymines (lower case letters). The 32*S* duplex is identical to the 32-mer released by the TstI REase: the letter P at both 5′ termini indicates the 5′ phosphate that would be left after REase cleavage.

Oligodeoxyribonucleotides, including those where an adenine in the TstI recognition sequence had been replaced by 6-methyladenine (m^6^A), were purchased from Eurofins MWG Operon. Pairs of complementary oligonucleotides were annealed to give the duplexes shown in Figure 1.

The plasmids pTST0, pTST1 and pTST2 were constructed from pDG5 (28), a molecule that lacks the cognate sequence for TstI but which was found to contain a secondary site for this enzyme (Supplementary Data). All three constructs lack the secondary site and carry, respectively, zero, one and two copies of the cognate recognition sequence for TstI. The TstI site in pTST1 and both sites in pTST2 are embedded in the same sequence as the oligoduplex 50*S*. The novel plasmids were used to transform *E. coli* HB101, the transformants grown in minimal media (with [*methyl*-^3^H]thymidine whenever radiolabelled DNA was sought) and the DNA purified by CsCl gradients as supercoiled (SC) monomers by previous methods (28–31).

### Molecular weight determinations

Analytical ultracentrifugation (AUC) employed a Beckman XL-A ultracentrifuge (22) to sediment to equilibrium TstI protein (0.1–0.3 mg/ml) in AUC buffer (20 mM Tris-HCl, pH 8.4, 100 mM KCl, 10 mM MgCl_2_) at 20°C. An initial *A*_280_ scan was made on reaching 3000 rpm and the protein concentration evaluated from the invariant absorbance across the cell. Further *A*_280_ scans were made after 18, 24 and 30 h at each of 5000, 6000 and 7500 rpm respectively. A final scan was made after 6 h at 40 000 rpm to record the baseline. Data were analysed by using ORIGIN to fit either single or multiple data sets to the equation for the radial distribution of a single species after sedimentation to equilibrium to yield the MW value that constituted the best fit to the relevant data set(s).

**Figure 2.**
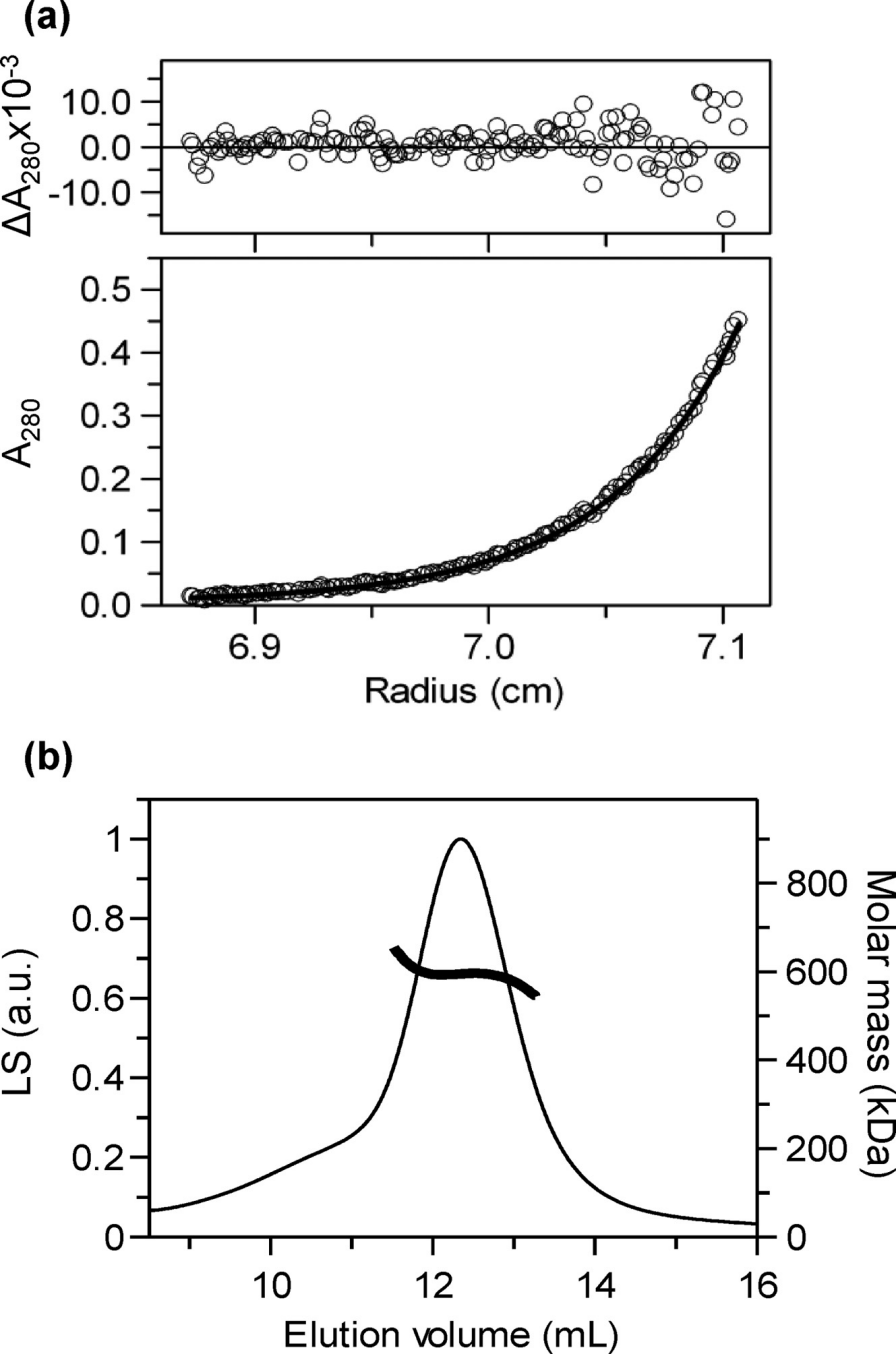
Molecular weights. (**a**) TstI RM protein (0.11 mg/ml) in AUC buffer was centrifuged at 6000 for 30 h at 20°C. The main panel shows the absorbance as a function of centrifugal radius. The line through the circles was obtained by a global fit to the equation for the sedimentation of a single ideal species, employing data from varied protein concentrations (0.11–0.32 mg/ml) and centrifugal velocities (5000–7500 rpm). The plot above the main graph displays the residuals between the global fit (which yielded a MW of 594 kDa) and the data from this particular experiment. (**b**) The trace shows the elution profile of the TstI protein in MALS buffer from a gel filtration column monitored by LS (Raleigh ratio in arbitrary units (a.u.), left-hand y-axis). Both LS and dRI (not shown) readings were taken at 0.5 s intervals throughout the chromatogram and each pair used to evaluate an independent value for MW: the thick line across the peak displays the individual MW values (right-hand y-axis) over this region. Across the segment of the elution profile selected (11.7–13.0 ml), a single molecular species was observed, as judged by a constant MW at an average of 594.2 kDa (±0.7%).

For multi-angle light scattering (MALS) measurements, a Superose6 10/300 column (GE Healthcare) was connected to an HPLC (Agilent Series 1200) and equilibrated overnight at a flow rate of 0.7 ml/min in MALS Buffer (as AUC buffer but with 2 mM CaCl_2_ in place of the MgCl_2_). An aliquot of TstI protein (≤100 μl) was loaded onto the column and the eluate passed through two detectors: a light scattering (LS) diode array (Dawn Heleos II) and a differential refractive index (dRI) detector (Optilab rEX), both from Wyatt, USA. Data from both detectors were recorded at 0.5 s intervals and MW values solved for each pair of points using the Wyatt software ASTRA 6. Mean MW values were then obtained over the selected region of the elution profile, as noted in Figure 2b.

### DNA cleavage reactions

DNA cleavage reactions were typically performed in 200 μl reactions containing 5 nM DNA and the requisite concentration of TstI in buffer R (10 mM Tris-HCl, pH 8.4, 100 mM KCl, 10 mM MgCl_2_, 0.1 mg/ml bovine serum albumin [BSA]), usually at 37°C. The DNA was the ^3^H-labelled SC form of either pTST1 or pTST2: pTST0 was used as a control for non-specific cleavage. Samples (10 μl) were taken before and at various times after adding the enzyme, quenched and analysed by electrophoresis through 1.2% agarose to separate the following (28,31,33): uncut SC substrate; open circle (OC) DNA cut in one strand at one or more sites; full-length linear (LIN) DNA with at least one DSB at one site; and, for the two-site plasmid pTST2, the products (L1 and L2) with at least one DSB at both sites. The DNA was detected with ethidium and the concentration of each form assessed by scintillation counting (22,28,37). [L1 + L2 denotes the mean of the measured concentrations of L1 and L2.]

The LIN, L1 and L2 species all exist in at least two states depending on whether the DNA is cleaved on one or both sides of the recognition site(s), but these states differ by only 32 bp and are inseparable on agarose. To measure the release of the 32-mer during plasmid cleavage, 20 μl samples were taken from the reactions at successive times, stopped and treated with proteinase K: half of each sample was then examined by electrophoresis through agarose as above; the other half by electrophoresis through 15% polyacrylamide in Tris-borate-EDTA in parallel with an aliquot of the 32*S* oligoduplex (22,37). The latter gels were stained with SYBR Safe (Invitrogen) and analysed in the PhosphorImager (22). The concentration of the 32-mer was determined relative to the known concentration of the 32*S* duplex loaded onto the same gel. The same procedure was used to follow the cleavage of the 211 bp polymerase chain reaction (PCR) product with one TstI site (Supplementary Figure S3).

### DNA methylation reactions

Methylation reactions were done by adding the requisite concentration of TstI protein to 100 nM DNA, either a plasmid or an oligoduplex, in buffer M (5 μM [*methyl*-^3^H]-SAM, 20 mM Tris-HCl, pH 8.4, 100 mM KCl, 2 mM CaCl_2_, 0.1 mg/ml BSA) at 37°C. Samples (20 μl) were taken before adding the enzyme and at various times afterwards. The subsequent processing of the samples—quenching the reactions by mixing with phenol/chloroform, removing the free ^3^H-SAM in a spin column and recording the level of ^3^H incorporation into the DNA by scintillation counting—were all as described before (38). The ^3^H-SAM (Perkin Elmer, UK) had been diluted with unlabelled SAM to give a specific radioactivity of 37 MBq/μmole: at this level, the incorporation of one methyl group per DNA molecule should result in 3300 dpm and the complete methylation of an UM site (one to each strand) 6600 dpm.

## RESULTS AND DISCUSSION

### Protein production

The TstI RM protein was generated from a synthetic gene designed to give the same amino acid sequence as the gene sequence from the thermophilic bacterium *T. scotoductus* but with optimal codons for expression in *E. coli* (Supplementary Figure S1). The gene was first cloned into an expression vector, pET15b, which yielded a His-tagged form of TstI with 20 extra amino acids at its N-terminus. The tagged protein was readily purified to homogeneity by Ni-affinity, heparin and size exclusion chromatography (Supplementary Data). However, the positive charge on the His residues in the tag could perturb the interactions of the protein with DNA. The extension encoded by pET15b contains a target sequence for thrombin, but thrombin cleaved His-tagged TstI at multiple locations (data not shown). The synthetic gene was therefore cloned in another vector, pET21a, to give the native protein without N- or C-terminal tags. Relative to the His-tagged form, purification of the native protein was more challenging, involved more stages and gave a lower yield due to losses at each stage (Supplementary Data). Even so, sufficient quantities of the native form were obtained for comparisons with the tagged protein. In all DNA cleavage reactions tested, the two proteins behaved identically (data not shown), so the His-tag is immaterial. All of the experiments described below used the tagged species, a polypeptide of 1271 amino acids (*M*_R_ 145,686), rather than the native protein of 1251 amino acids, and the name TstI will refer here to the tagged protein.

### Molecular weight determination

Gel filtration of AloI, a single-polypeptide Type IIB system closely related to TstI (26), had yielded an MW approximately 4-fold larger than that of the peptide chain, suggesting a tetramer (25). However, MW values from gel filtration depend not only on the mass but also the shape of the protein and can deviate from the true MW. Two shape-independent methods were used here to evaluate the MW of the TstI protein in solution: AUC to sedimentation equilibrium and MALS (Figure 2).

A range of concentrations of the TstI protein were sedimented for 18–30 h at varied centrifugal velocities: at each speed, no change in the radial distribution of the protein was observed after 24 h, indicating that equilibrium had been reached. The data at each protein concentration and at each velocity were fitted to the equation for the sedimentation of a single ideal species to find the best MW for each set. The best fits to the separate sets all fell in a narrow zone, 602 (±28) kDa. No systematic variations were seen across the span of concentrations tested, thus excluding the possibility of protein association events over this range. The complete series of data sets were then subjected to a global fit to evaluate a single MW value (Figure 2a). The best fit to the complete set was with an MW of 594 kDa, which matches closely the theoretical MW for a tetramer of the His-tagged TstI polypeptide, 583 kDa.

The MW of the TstI protein in solution was also determined by MALS (Figure 2b). Size exclusion chromatography of the TstI protein revealed a single peak of material in the elution profile. MW values were obtained from each pair of LS and dRI readings recorded during the chromatogram: samples across the peak gave a constant MW, indicating a single homogeneous species with an average at 594 kDa, matching that from AUC.

A tetrameric structure for the TstI protein poses several questions about its mode of action. Many Type II REases are tetramers that cleave DNA at two copies of a palindromic DNA sequence (29,35,39). Tetramers of this sort, such as SfiI or Cfr10I, resemble ‘dimers-of-dimers’: two subunits bind symmetrically to one copy of the DNA and cut both strands at that copy, while the other two subunits lie back to back with the first pair and interact with a second copy of the DNA. Each subunit contacts only half of the target sequence but the polypeptide chain of TstI has in its DNA-binding domain the components for sensing both segments of its bipartite recognition sequence (26), so a single subunit could span the full length of the sequence. Each subunit might thus bind a separate copy of the recognition site, which could lead to the tetramer carrying four DNA segments. In addition, the TstI tetramer is likely to contain one active site for phosphodiester hydrolysis in each subunit. The four together may allow it to make two DSBs at the same time.

There exist at least two examples of homotetrameric REases that act simultaneously at two copies of an asymmetric sequence, BspMI amd MspJI (41,42), both Type IIS enzymes (8). Both employ the active sites from all four subunits to make two DSBs, one at each copy of the recognition site. Moreover, while the catalytic centres from two subunits of the MspJI tetramer become juxtaposed to make a DSB at one site, each subunit possesses an independent DNA-binding domain so the protein can bind four DNA segments at the same time. Both BspMI and MspJI cut the DNA at fixed distances away from their sites but only downstream of their asymmetric sites, in the IIS style (8). TstI, on the other hand, can make two DSBs at each site, one upstream and one downstream of the site. There are numerous possibilities for how TstI might deploy its four catalytic centres, especially if it needs to interact with at least two copies of its recognition sequence at the same time (see below). One is all four catalytic centres are positioned towards one recognition sequence, in which case a DNA with one TstI site might be converted directly to the final product with two DSBs at that site, with concomitant release of the 32-mer. The converse would be that the DNA bound to one subunit is attacked only by the catalytic centre in that subunit to create a single nick on one side of that site: multiple cycles of association and dissociation would then be needed to cut all four bonds before eventually releasing the 32-mer.

### DNA cleavage: multiple turnovers

**Figure 3.**
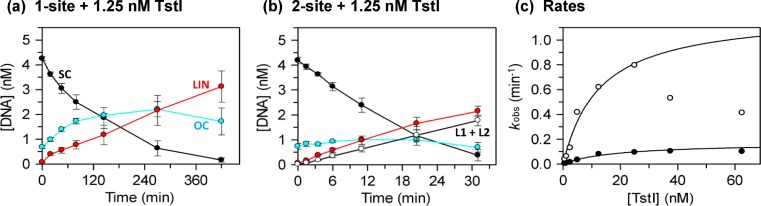
Catalytic reactions of TstI REase. (**a**) and (**b**) The reactions, in buffer R at 37°C, contained 1.25 nM TstI tetramer and 5 nM ^3^H-labelled SC DNA, either pTST1 (a) or pTST2 (b). The plasmids pTST1 and pTST2 carry respectively one and two copies of the recognition sequence for TstI. Samples were taken at the indicated times (note the different time scales) and analysed as in Materials and Methods to give the concentrations of the following forms of the DNA: SC substrate, in black; nicked OC DNA, in cyan; the LIN form with at least one DSB at a single TstI site, in red; and from the two-site plasmid the products with at least one DSB at both sites (L1 + L2), unfilled circles. Means of triplicate measurements are shown: error bars denote standard deviations. (**c**) Reaction on pTST1 and pTST2 was carried out as above with varied concentrations of TstI. First-order rate constant (*k*_obs_) was evaluated by fitting the decline in the concentration of the SC substrate with time to an exponential decay and the values of *k*_obs_ plotted against the enzyme concentration: reactions on pTST1, black circles; on pTST2, unfilled circles. The lines drawn reflect the best fits to rectangular hyperbolas: for pTST1, the fit to all enzyme concentrations tested gave *k*_max_ = 0.17 min^−1^ and *K*_1/2_ = 19 nM; for pTST2, the fit was limited to enzyme concentrations ≤25 nM and gave *k*_max_ = 1.22 min^−1^ and *K*_1/2_ = 13 nM.

The BcgI REase cleaves plasmids with two cognate sites by means of reactions *in cis*, bridging sites in the same DNA molecule, but it works on one-site plasmids *in trans*, synapsing sites on separate DNA molecules (37). However, its cleavage of the one-site DNA was not only much slower than the two-site substrate (27,28) but also required a molar excess of enzyme over DNA, the hallmark of an enzyme operating stoichiometrically rather than catalytically (22). To see if the REase component of the TstI protein behaved similarly, its reactions on plasmids with one and two copies of its cognate sequence, pTST1 andpTST2 respectively, were studied at a fixed DNA concentration but with enzyme concentrations varying from below to above that of the DNA (Figure 3).

In reactions containing TstI protein at lower concentrations than the DNA, the SC forms of both one- and two-site plasmids were converted in their entirety to either nicked or linear species. Since one molecule of TstI tetramer can cut several molecules of DNA, it must carry out multiple turnovers. The REase activity of TstI is therefore not limited to acting stoichiometrically but instead functions catalytically. Nevertheless, at sub-stoichiometric concentrations of enzyme, the reaction on the one-site DNA was extraordinarily slow. For that shown in Figure 3a, with a 4-fold molar excess of DNA over protein, it took ∼7 h for all of the SC substrate to be cut. Even after 7 h, a substantial fraction of the one-site plasmid had only been nicked: much of it had yet to be converted to the linear product(s) with DSB(s). In addition, though the rate of utilisation of the two-site substrate was faster than that of the one-site DNA, it too yielded initially a mixture of products: some with DSBs at both sites, others cut at only one site (Figure 3b).

In reactions on both one- and two-site substrates, the decline in the concentration of the SC DNA followed an exponential progress curve that was fitted to give a first-order rate constant *k*_obs_, regardless of whether the TstI concentration was above or below that of the DNA (Figure 3c). On the one-site plasmid, the values for *k*_obs_ increased with increasing enzyme concentrations in a hyperbolic manner to a maximal rate (*k*_max_), presumably at saturation of the substrate with excess enzyme. The enzyme concentration for the half-maximal rate, *K*_1/2_ = 19 nM, corresponds to the equilibrium dissociation constant of the active enzyme–substrate complex to free components. It should however be noted that the values for *k*_obs_ from multiple-turnover reactions, with excess DNA, reflect different parameters compared to those from single-turnover reactions with excess enzyme: the former spans the complete turnover ending with enzyme–product dissociation, while the latter relates to the formation of the first enzyme–product complex in the pathway, in this case the TstI protein bound to nicked DNA. Consequently, the observation that both multiple- and single-turnover reactions matched the same hyperbolic function shows that the rate-limiting step for the turnover of TstI must be at or before the formation of that first enzyme–product complex.

On the two-site plasmid, pTST2, increases in the enzyme concentration initially resulted in increasing reaction rates, again in a hyperbolic manner with a similar *K*_1/2_ as above but with a ∼10-fold higher *k*_max_ (Figure 3c). But further increases in concentration reduced the reaction rates to give *k*_obs_ values below those expected by extrapolating the hyperbola. The rate of utilisation of the two-site plasmid was nevertheless much faster than that of the one-site DNA at all TstI concentrations tested. Hence, as with virtually all other Type IIB proteins (17,28), the TstI REase needs to interact with two sites for full activity. Proteins that need two sites nearly always prefer them in the same DNA chain over those on separate DNA molecules (34,39). In this particular case, this conclusion is validated by the reduction in reaction rate on the two-site substrate at elevated enzyme concentrations: the optimal reaction rate presumably involves a single tetramer of TstI spanning two sites *in cis*, looping out the intervening DNA, but excess enzyme over sites will lead to tetramers binding to both sites and so blocking the looping event. This had been seen before with other REases that cleave two-site substrates at reduced rates at high enzyme concentrations (33,36).

### DNA cleavage: single turnovers

**Figure 4.**
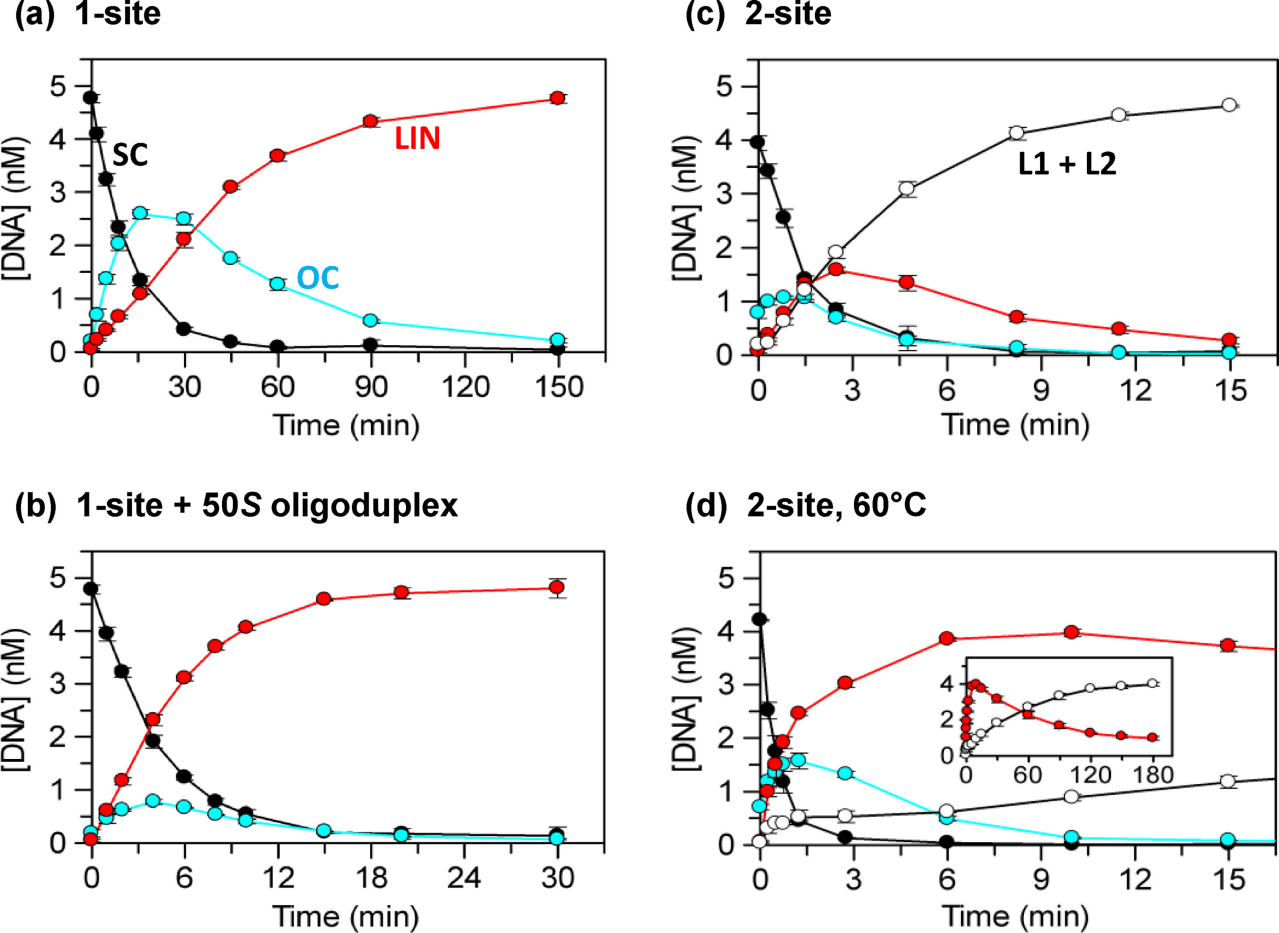
Single-turnover reactions of TstI REase. The reactions all contained 12.5 nM TstI tetramer and 5 nM ^3^H-labelled plasmid, as indicated below, in buffer R: (**a**) the reaction on the one-site plasmid pTST1 at 37°C; (**b**) the reaction on pTST1 at 37°C (as (a)) but now supplemented with 50 nM 50*S*, an oligoduplex with the TstI site; (**c**) the reaction on the two-site plasmid pTST2 at 37°C; (**d**) the reaction on pTST2 (as (c)) but now at 60°C. Samples were taken at the indicated times (over varied time scales) and analysed as before to give the concentrations of the following forms of the DNA: SC substrate, black circles; nicked OC DNA, in cyan; LIN DNA with at least one DSB at a single site, in red; and from the two-site plasmid the products with at least one DSB at both sites, L1 + L2, unfilled circles. The plots show triplicate measurements of the concentrations of each form as a function of time: error bars denote standard deviations. The insert in (d) shows the LIN (red circles) and the L1 + L2 (unfilled circles) forms over an extended time base.

**Figure 5.**
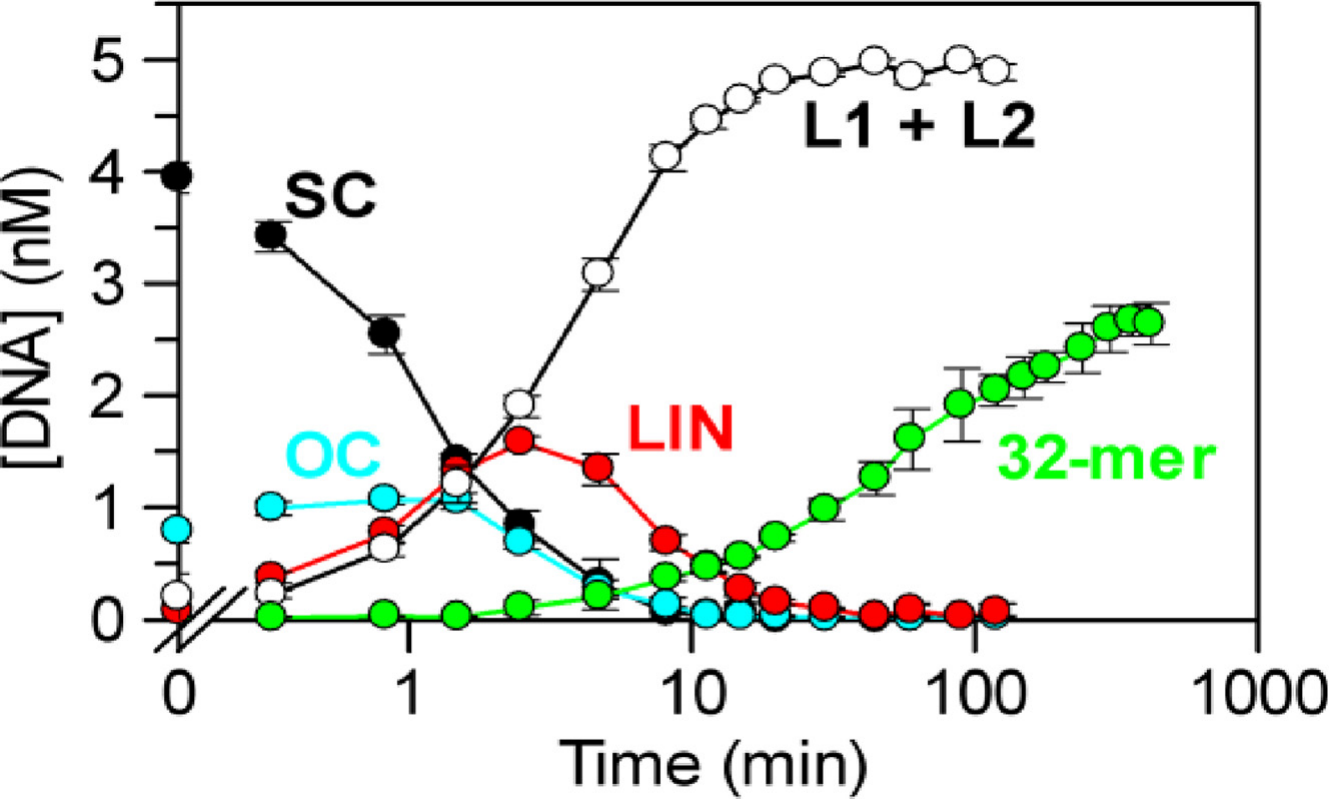
DNA excision by TstI REase. The reaction contained 5 nM SC pTST2 (^3^H-labelled) and 12.5 nM TstI in buffer R at 37°C (i.e. identical to that in Figure 4c). Samples were taken at the indicated times, quenched and then divided into two equal volumes: one for analysis by electrophoresis through agarose, the other through 15% polyacrylamide. The agarose gels were processed as above to give the concentrations of: SC DNA, black circles; OC DNA, in cyan; LIN DNA, in red; and L1 + L2, unfilled circles. [Data from the agarose gels are shown for 2 h: no further changes were observed at >2 h.] Polyacrylamide gels were processed as in Materials and Methods to give the concentration of the 32-mer (green circles), relative to a known concentration of the 32*S* oligoduplex applied to the same gel. The change with time in the concentrations of all of the species noted above are plotted on a logarithmic time scale but as pTST2 has two TstI sites, each of which can release the 32-mer, the concentration of the 32-mer shown is half of that measured. [Complete cleavage of 5 nM plasmid, on both sides of both sites, would be noted here as 5 rather than 10 nM 32-mer.] Data reflect triplicate repeats of both gels; error bars mark standard deviations.

Under multiple-turnover conditions, the rate of DNA cleavage by TstI was so slow that it would have taken an inordinate length of time to observe the complete progress of the reaction through to its final products with DSBs at each recognition site. Consequently, DNA cleavage by the TstI RM protein was monitored mainly under single-turnover conditions (Figure 4), with a protein concentration above that of recognition sites on the DNA but below the level that led to diminished rates on the two-site substrate (Figure 3c). Under these conditions, the profile of the reaction on the SC plasmid with one TstI site (Figure 4a) showed the classical signature of a sequential two-step process (43), an A → B → C pathway with equal rates at each step. Hence, the enzyme first cuts one strand of the one-site DNA to convert the SC substrate to the nicked OC form and then, at the same rate, the second strand opposite the nick to give the LIN product with at least one DSB at the TstI site. Whether the enzyme has cut the DNA on both sides of its site is addressed below (Figure 5 and Supplementary Figure S3).

The reaction of TstI on the one-site plasmid was also studied in the presence of an oligoduplex that carries the recognition sequence for TstI (Figure 4b). The specific duplex used here, 50*S*, is a full substrate for TstI, with not only the recognition sequence but also upstream and downstream cleavage loci (Figure 1). The SC plasmid was cleaved more rapidly in the presence of the duplex (Figure 4b) than in its absence (note the different time scales in Figures 4a and b). In addition, the reaction containing the duplex proceeded in a more concerted manner than that in its absence. Instead of cutting one strand at a time as had been the case in the absence of the duplex, its presence resulted in almost all of the SC plasmid being converted directly to the LIN product with at least one DSB break at the recognition site, without extensive accumulation of nicked species.

Additional experiments employed varied concentrations of the 50*S* duplex (Supplementary Figure S2). The extent of plasmid cleavage initially increased with increasing concentration of 50*S* up to a maximum (at the concentration used in Figure 4b), but further increases then reduced the extent of plasmid cleavage to levels below that in the absence of 50*S*. Neither a 50 bp non-specific DNA lacking the recognition sequence (50*NS*; Figure 1) nor a shortened duplex carrying the central 20 bp from 50*S* (i.e. with the recognition sequence but not the cleavage loci) had any effect on the extent of plasmid cleavage (Supplementary Figure S2), even though both were found in gel shifts to bind to the protein (data not shown). Enhanced cleavage of the plasmid with one TstI site thus requires a second substrate with both recognition and cleavage loci rather than a duplex with an uncleavable recognition site as is the case for many restriction enzymes that need two sites (32).

The enzyme bound to its site on the plasmid thus needs to interact with a second substrate and for steric reasons, this will occur more readily with a short linear duplex than a large SC plasmid (29,34). The synaptic complex between the plasmid and the duplex appears to have a sufficiently long lifetime to allow the enzyme to cut both DNA strands at the plasmid's recognition site (Figure 4b) while that involving two molecules of the plasmid will, for entropic reasons, have a shorter lifetime and so dissociate before cutting both strands (Figure 4a). The reduced cleavage of the plasmid with high concentrations of the 50*S* duplex is doubtless due to the enzyme binding two molecules of the duplex instead of one duplex and one plasmid.

Single-turnover conditions were also employed to monitor the complete time course of the cleavage of a plasmid with two TstI sites, pTST2 (Figure 4c): the protein concentration used here was the same as that for the one-site DNA, still in excess over recognition sites on the DNA though at a different ratio. As noted above (Figure 3c), the rate on the two-site plasmid was considerably faster than on the one-site DNA. The two-site DNA was also cleaved in a more concerted manner than the one-site substrate in the absence of specific duplex. Rather than proceeding first to the nicked OC form and then to the LIN species with a DSB at the site, the reaction on the two-site substrate with excess enzyme yielded very little OC DNA and only a low level of the LIN form cut in both strands at a single site. Instead, the majority of the two-site DNA was converted directly to the species with at least one DSB break at both sites, the two linear fragments L1 and L2. The addition of the specific 50*S* duplex failed to enhance—and instead inhibited—cleavage of the two-site DNA (Supplementary Figure S2). The faster rate on the two-site DNA and the lack of activation by the duplex show that TstI cleaves a DNA with two sites by spanning sites *in cis* on the same molecule of DNA rather than binding to sites *in trans* on separate DNA molecules. Furthermore, the failure of the duplex to enhance cleavage of the two-site plasmid indicates that the TstI tetramer acts at only two recognition sites at a time even though it might be capable of binding four.

All of the DNA cleavage studies described above were carried out at 37°C. The previous studies on the two-polypeptide Type IIB systems, including BcgI (20,22,27,37), and those on the single-polypeptide IIB proteins had all been done at 37°C (24,25,28). TstI reactions at 37°C can thus be compared directly with other Type IIB proteins. Nevertheless, TstI is from a thermophilic organism that grows at 70°C (2), and its behaviour at its native temperature had yet to be characterised. To examine this point, stability trials were conducted by incubating the TstI protein in buffer R for 3 h at various temperatures before adding the pTST2 plasmid and then monitoring cleavage at 37°C: full activity was retained at all temperatures ≤60°C (data not shown). The reaction of TstI on the two-site substrate at an elevated temperature was then monitored at 60°C (Figure 4d). At this temperature, the concentration of the SC substrate declined more rapidly than at 37°C but instead of the concerted process seen at 37°C that led directly to the linear fragments (L1 and L2) with DSBs at both sites (Figure 4c), the primary product from the 60°C reaction was the LIN DNA with a DSB break at only one site (Figure 4d). The LIN form was subsequently cleaved at its intact recognition site at a very slow rate to give the L1 and L2 products (inset to Figure 4d). Though TstI comes from a thermophilic organism and so might be expected to display its maximal activity at elevated temperatures, it takes much longer to cut both sites on a two-site DNA at 60°C than at 37°C. The synaptic complex spanning two TstI sites *in cis* probably has a shorter lifetime at 60°C than at 37°C, with the result that at 60°C it falls apart before the enzyme can cut both sites.

### Excision of the recognition site

The Type IIB REases cut DNA on both sides of their recognition sites to excise a small fragment carrying the site; in the case of TstI 32 nt long in both strands (2). In the above assays, the SC plasmids were separated from the various reaction products by electrophoresis through agarose. However, an additional 32 bp at the end of a kb-sized fragment will not cause any detectable change in mobility so it is impossible to tell whether the linear species observed in the agarose gels carried DSBs on one or on both sides of the site(s). Further reactions of TstI on the two-site plasmid pTST2 were therefore carried out to measure in parallel the formation of the linear products cleaved in both strands at one or more loci and the release of the 32-mer cut on both sides of a site. Samples were taken from the reactions, quenched and then one half was analysed on agarose while the other half was applied to polyacrylamide to capture the 32-mer (Figure 5). The analysis on agarose was as above (viz. Figure 4c), albeit now presented on a logarithmic scale: again, essentially all of the SC DNA was converted within 20 min to the two linear products (L1 and L2) with at least one DSB at both sites. Yet after 20 min, only 20% of the recognition sites had yielded the 32-mer and even after 200 min, the 32-mer had been released from less than 50% of the recognition sites (Figure 5).

The TstI REase thus makes its first DSB on one side of its recognition sequence much more rapidly than the second side. In this respect, TstI differs markedly from the BcgI REase (37): BcgI generates the linear products seen on agarose, with at least one DSB at each site, at the same time as the excised product with two DSBs at each site. Moreover, on a DNA with two cognate sites, BcgI reactions yield directly the excised products from both sites, without liberating intermediates cut at only one site. Hence, while BcgI can cleave eight phosphodiester bonds in a single synaptic complex across two sites, the same is not the case for TstI.

The plasmid with one TstI site also yielded the 32-mer but at too slow a rate to measure precisely. [The initial cleavage of the one-site plasmid is slow (Figure 4a) and the liberation of the 32-mer even slower (data not shown).] The formation of the 32-mer from a one-site substrate was therefore monitored on a linear DNA that had been generated by PCR amplification of a 211 bp segment of pTST1 spanning its TstI site. The 211 bp DNA, named ABC where section B denotes the 32-mer and A and C the peripheral segments (Supplementary Figure S2), was designed so that DSBs on the left of the site (to give A + BC), on the right (AB + C) and on both sides (A, B, C) all gave unique fragments that could be separated from each other by electrophoresis through polyacrylamide. The 211 bp PCR fragment was cleaved more rapidly than the one-site plasmid (Supplementary Figure S3), as expected since proteins that bridge two sites on separate molecules do so more readily on linear than on SC DNA (29,34). The linear DNA was however cut first on just one side of the site, with roughly equal probabilities for the left or the right of the site, as judged by the initial rates of formation of the BC and the AB products. Only later in the reaction were the AB and BC intermediates cleaved again to release B, the 32-mer.

Thus on both one- and two-site substrates, the TstI REase cuts on both sides of its site in separate reactions: after cutting on one side, it then cuts on the other at a much slower rate. The difference in rate cannot be assigned to the enzyme preferring one side over the other since both sides were cleaved more or less equally in the initial reaction. Instead, TstI finds it difficult to trim the 32-mer off a linear product already cut on one side of the site.

### Parallel cleavage and methylation

The methylation co-factor SAM is required for both the MTase and REase activities of the BcgI RM protein (19,20,40) but the BcgI MTase is inactive at UM sites (38). Hence, in the presence of both SAM and Mg^2+^ as would be the case *in vivo*, UM sites are always restricted by BcgI rather than modified. TstI, on the other hand, does not require SAM in its REase role, which leaves open the question of how TstI behaves in reactions containing both SAM and Mg^2+^.

**Figure 6.**
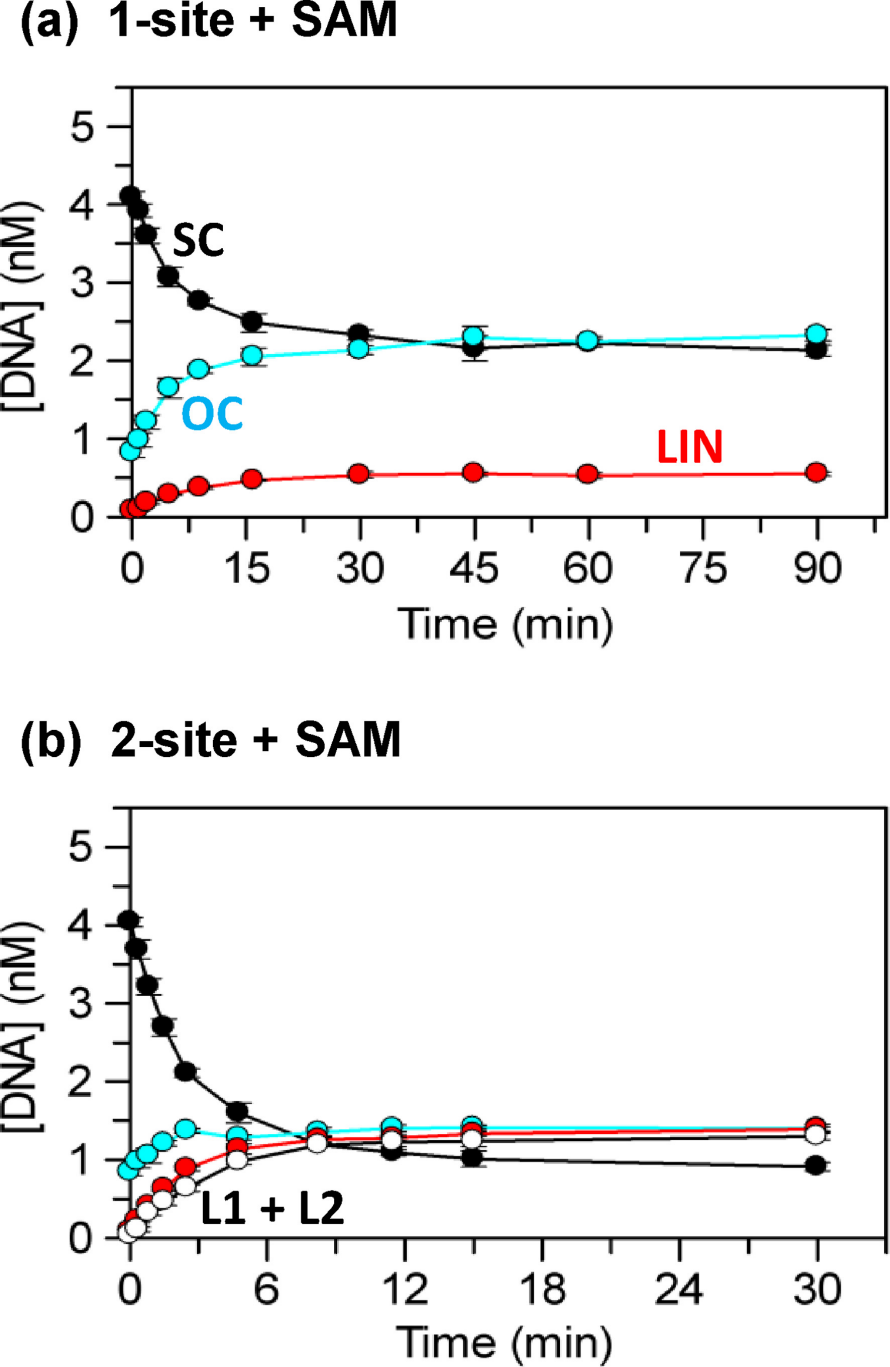
Parallel restriction and modification. The reactions shown in (**a**) and (**b**) were the same as those in Figures 4a and c respectively (12.5 nM TstI tetramer and 5 nM ^3^H-labelled SC plasmid [pTST1 in (a), pTST2in (b)] in buffer R at 37°C) except for the addition of 20 μM SAM. The reactions were analysed as in Figure 3 to determine the concentration of each DNA species, plotted here against time: SC DNA, in black; OC DNA, in cyan; LIN DNA, in red; and in (b) alone, L1 + L2, unfilled circles. Triplicate means are shown, with standard deviations.

The ability of TstI to cleave plasmids with one or two recognition sites was tested with both co-factors present (Figure 6). The Mg^2+^-dependent cleavage of the one-site plasmid started off at much the same rate in the presence of SAM as in its absence, until about half of the SC DNA had been cut. But the reaction then ground to a halt with no further cutting of the remaining DNA (Figure 6a). This behaviour suggests that the REase and MTase components of the TstI protein have similar activities at the single UM site on the plasmid, with the result that about 50% of the sites are cleaved by the REase while 50% are modified by the MTase, after which they can no longer be cleaved. Similar behaviour was also observed on the plasmid with two TstI sites except that this time about 75% of the DNA was cleaved by the REase while 25% remained resistant to cleavage (Figure 6b). The increase in the extent of cleavage can be accounted for by the TstI protein having a higher REase activity on two-site substrates.

These experiments show that in the presence of both MTase and REase co-factors, SAM and Mg^2+^ respectively, the TstI RM protein can either methylate or hydrolyse an UM site, with approximately equal efficiencies. It might thus seem that the TstI RM system would be incompetent at restricting foreign DNA *in vivo* as naive DNA that lacked TstI methylation could be modified rather than restricted as it enters a cell carrying this RM system. However, to evade restriction by the TstI RM protein, an unexposed DNA with multiple TstI sites must be methylated at every site before the REase had cleaved any one site. For example, given equal REase and MTase activities at an individual site, the probability of a DNA with 10 TstI sites becoming methylated at all 10 sites before any cleavage event is 0.5^10^; i.e. 1.10^−3^. Moreover, a DNA with multiple TstI sites is cleaved more rapidly by the TstI REase than DNA with one site (Figures 3 and 4), which further favours restriction over modification (Figure 6b). Hence, despite its significant MTase activity at UM sites, TstI still ought to be able to restrict DNA *in vivo*.

### Methylation of plasmids

The MTase activity of the TstI RM protein was studied in isolation from its REase by employing Ca^2+^ in place of Mg^2+^ and unlabelled DNA in place of the ^3^H-labelled substrates used above. As with many other REases (44), Ca^2+^ completely blocked DNA cleavage by TstI (data not shown). The extent of methylation was then measured from the incorporation of radiolabel from [*methyl*-^3^H]SAM into the DNA (38). Methylation assays first used the SC plasmids pTST0, pTST1 and pTST2, which carry respectively zero, one or two TstI sites. These assays thus ought to reveal whether the TstI MTase needs to interact with two copies of its recognition sequence as had been the case for its REase. They ought also to reveal the validity of the above proposal (Figure 6), namely that the TstI RM protein can modify UM sites at much the same rate as cutting them.

**Figure 7.**
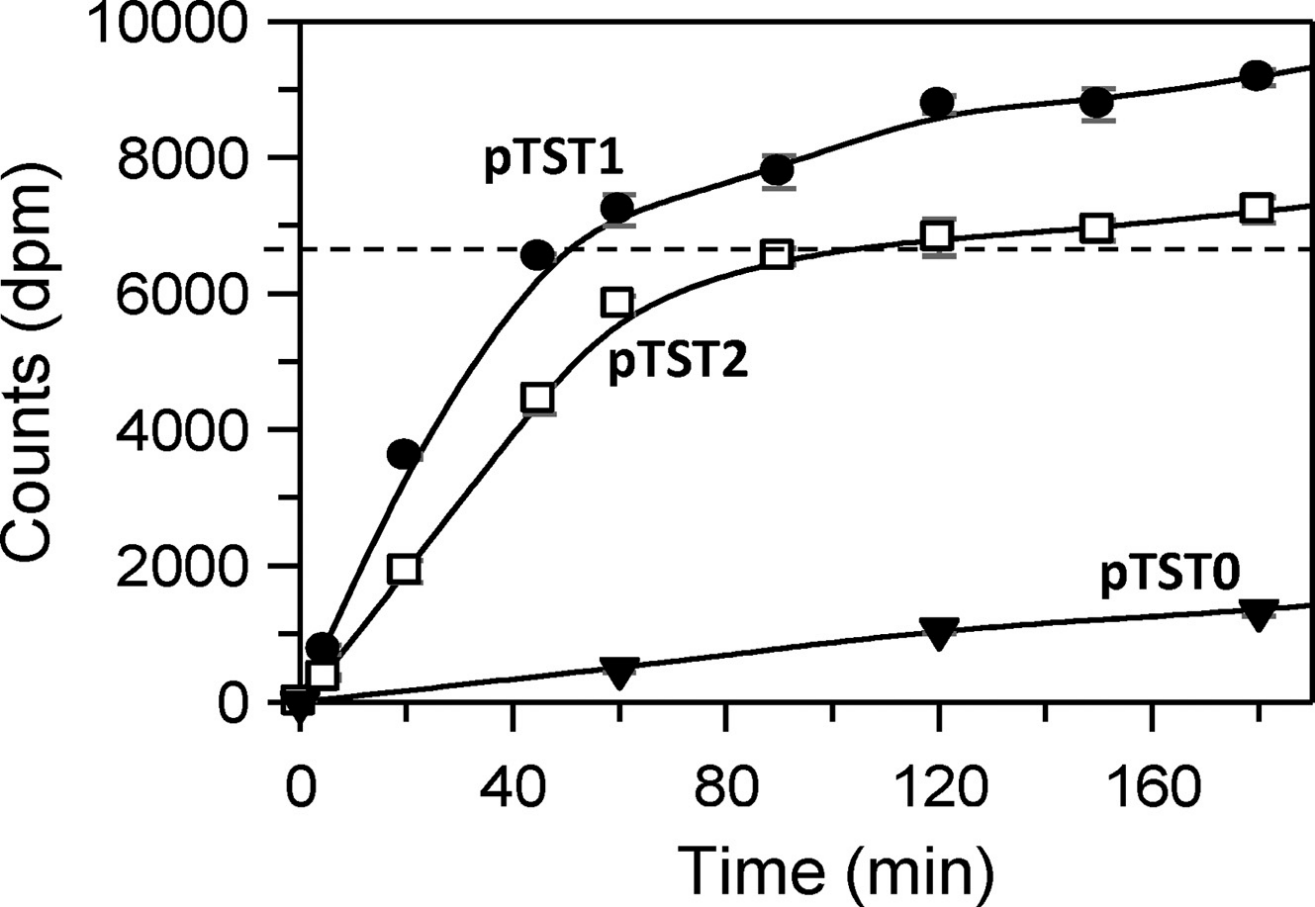
Methylation of plasmids. Reactions, in buffer M at 37°C, contained 100 nM TstI protein and 100 nM SC plasmid. Samples were withdrawn from the reactions at the times indicated, stopped immediately and the level of radioactivity transferred from the [*methyl*-^3^H]SAM in buffer M to the DNA was measured as described in Materials and Methods. The dashed horizontal line represents the expected dpm reading from the incorporation of two methyl groups per recognition. The DNA was one of the following: pTST0 with no TstI recognition sites, filled inverted triangles; pTST1, with one TstI site, filled circles; pTST2, with two TstI sites, unfilled squares (the dpm values shown here are the experimental values divided by two to allow for direct comparison with the one-site plasmid). Means of three repeats are shown with error bars noting standard error of the mean.

The plasmid with one TstI site incorporated radiolabel at a relatively rapid rate, to an initial level corresponding to two methyl groups per DNA molecule (Figure 7), but further incorporation then continued at a slower rate. However, the slower rate matched the rate on the plasmid with no TstI sites (Figure 7) so it can be assigned to methylation at non-canonical sites. Once corrected for this background, the initial phase reflects the methylation of the UM TstI site, presumably one methyl group to each strand. At comparable enzyme:DNA ratios, specific methylation in the presence of Ca^2+^ (Figure 7) occurred at a similar rate to the cleavage reaction with Mg^2+^ (Figure 4a). Hence, the TstI RM protein can indeed methylate UM sites at much the same rate as cutting them. The one-polypeptide Type IIB protein TstI differs markedly in this respect from the two-chain system BcgI which can only methylate HM sites (38).

The plasmid with two TstI sites incorporated, as expected, twice as much radiolabel as the one-site plasmid but, once normalised for the number of sites, the methylation rate on the two-site plasmid was similar to that on the one-site DNA (Figure 7). While the TstI RM protein had cleaved the plasmid with two sites more rapidly than the one-site DNA, its MTase operates equally well on one- and two-site plasmids. Methylation thus presumably occurs independently at each copy of the recognition site. In this aspect, TstI behaves similarly to BcgI, which also needs two copies of its cognate site for its REase activity but just one for its MTase function (37,38).

A significant rate of methylation was observed on the plasmid with no TstI sites (Figure 7). It has been reported before that the MTases from many RM systems transfer methyl groups to non-canonical sequences, in some instances with only marginally reduced efficiencies relative to the canonical site (45,46). The TstI MTase appears to be another example of this behaviour. Most Type II RM systems encode separate proteins for restriction and modification, so that the abilities of each protein to discriminate against non-canonical sequences are usually unrelated to each other: the REase needs to show a high level of discrimination, to avoid potentially lethal cleavages at non-canonical sites, but additional methylation events may be innocuous. In contrast, the TstI RM system features a single protein with the same DNA recognition domain for both its REase and MTase roles. The catalytic activity of TstI MTase must therefore be less tightly coupled to the recognition of cognate DNA than its REase activity.

### Methylation of oligoduplexes

A series of oligoduplexes (Figure 1) were also tested as substrates for the TstI MTase. These consisted primarily of a set of 50 bp duplexes, each with a centrally located TstI site and both upstream and downstream loci for cleavage by the REase, but with varied methylation patterns at the site: an UM duplex lacking TstI methylation in either strand, 50*S*; HM duplexes with 6mA in place of the target adenine in either top or bottom strand, $50S^M$ and $50S_M$ respectively; a FM DNA with 6mA in both top and bottom strands, $50S_M^M$. The HM duplexes ought to be able to accept a methyl group on their unmodified strands but the FM DNA already carries 6mA in both strands of the site so any methylation of this duplex must be non-specific. In a further duplex, 50*NS*, the TstI site was disrupted by A→T mutations at both target adenines: this DNA serves as a non-specific control that, like the $50S_M^M$ derivative, cannot be methylated at the recognition site. Another duplex, 32*S*, is identical to the 32-mer released from the recognition site in 50*S* (or pTST1) when TstI makes DSBs on both sides of the site. The latter should establish whether the protein continues to methylate the site after its excision from the DNA.

**Figure 8.**
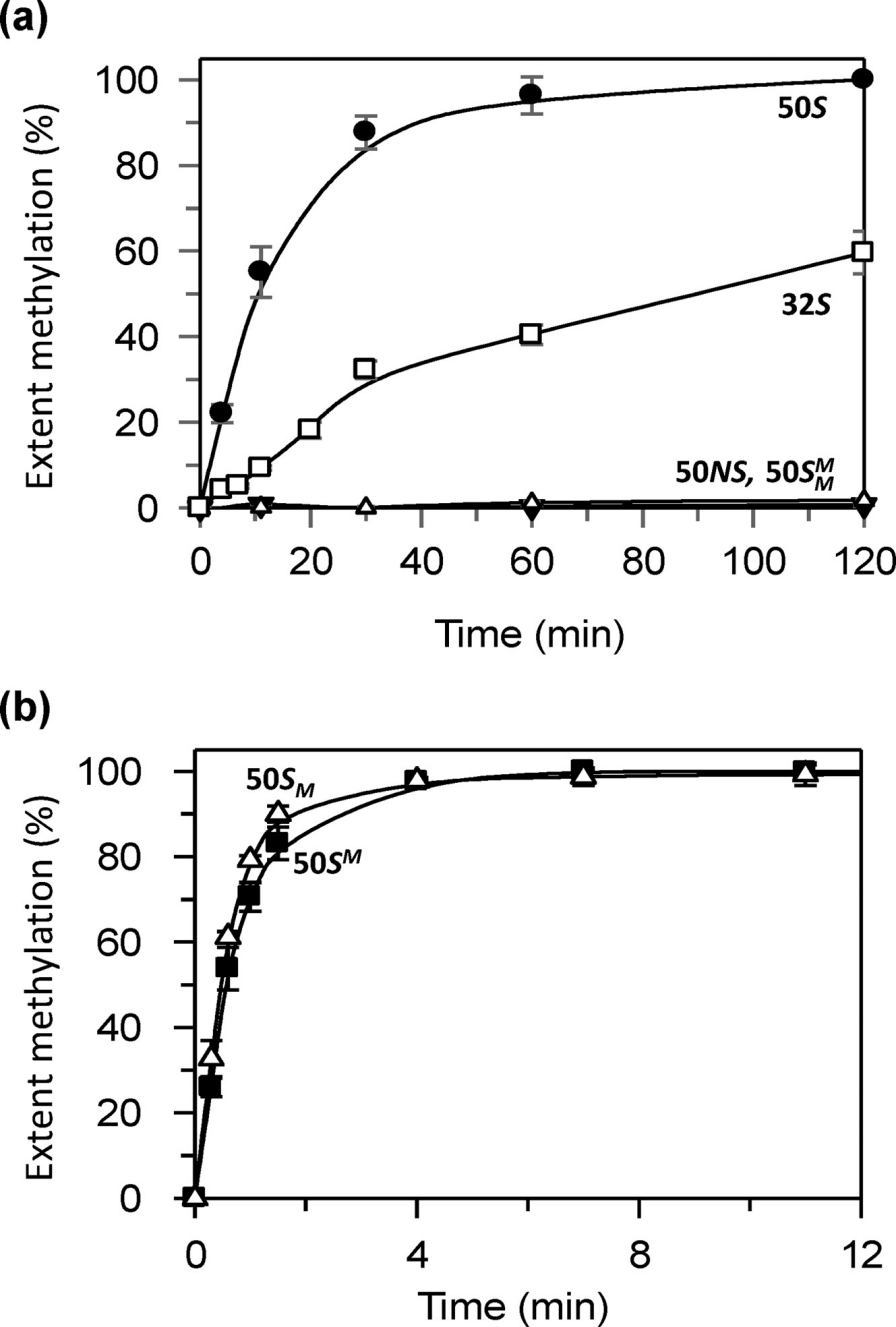
Methylation of oligoduplexes. Reaction, in buffer M at 37°C, contained 500 nM TstI protein and one of the oligoduplexes (from Figure 1) indicated below at 100 nM. Samples were withdrawn at the times indicated, stopped and the extent of methylation measured as in Materials and Methods. Panel (**a**) shows over a 120 min time scale the reactions on: 50*S* (the unmodified substrate), filled circles; 32*S* (equivalent to the UM product excised by the REase), unfilled squares; 50*NS* (a non-specific variant of 50*S*, with substitutions at the adenines modified by TstI), unfilled triangles; $50S_M^M$ (the FM form of 50*S* with 6mA residues in both strands), filled inverted triangles. Panel (**b**) shows over a 12 min time scale the reactions on: $50S^M$ (a HM form of 50S, modified in the top strand), filled squares; $50S_M$ (the HM form modified in the bottom strand), unfilled triangles. Extents of methylation are given: in (a), as a percent of that for the transfer of two methyl groups to an UM site; in (b), as a percent of that for one methyl group to a HM site. Data points are averages of three repeats with error bars for standard error of the mean.

The unmodified 50 bp duplex with both recognition and cleavage loci, 50*S*, was readily methylated by the TstI RM protein (Figure 8a). The reaction end-point corresponded to the transfer of two methyl groups to this DNA, one to each strand at the UM site. No significant transfer occurred to either the non-specific duplex lacking the target adenines (50*NS*) nor to the duplex already methylated at both adenines ($50S_M^M$). The lack of transfer to the non-specific duplex (Figure 8a) differs from the plasmid without a TstI site (Figure 7) but this is most likely a simple consequence of DNA length: the 3.9 kb plasmid has many more alternate sequences than the 50 bp duplex. Methyl transfer to the 50*S* duplex occurred at much the same rate as those to the one- and two-site plasmids.

The 32-mer was also methylated by the TstI RM protein, though at a slower rate than the 50 bp duplex (Figure 8a). Hence, TstI could in principle continue to methylate the 32-mer after excising it from the remainder of the DNA, as had been noted for BcgI (19). However, BcgI cuts on both sides of its site at the same time so the excised fragment is released concomitantly with the DSBs, presumably as an UM fragment which could perhaps be modified later. In contrast, it is doubtful if the TstI REase would ever release an UM 32-mer that could become available for subsequent modification by the TstI MTase. In the absence of SAM, the REase domain of the TstI protein excises the 32-mer much more slowly than it makes its first DSB break at a site (Figure 5), while the rate of methylation of the 32*S* duplex in the presence of SAM (Figure 8a) suggests that the site will often be methylated before the second DSB. This would prevent that scission from ever happening. The excision event probably plays little if any role in the restriction of foreign DNA by the TstI system, which must instead rely primarily on the initial DSBs on one side of each site.

The above methylation studies had all been carried out with a large excess of TstI protein over recognition sites on the DNA to ensure complete methylation of the DNA. To see if the MTase activity of the TstI protein could function catalytically, methylation of the 50S duplex was studied at varied concentrations of the protein tetramer from above to below that of the DNA (Supplementary Figure S4). Lower concentrations of the TstI MTase gave lower methylation rates but sub-stoichiometric levels of the protein still gave reactions progressing towards full methylation of the substrate. The TstI protein can thus operate catalytically and carry out multiple turnovers in both its MTase and REase reactions. Conversely, BcgI functions catalytically only in its MTase reaction, though even then requiring excess protein to transfer a single methyl group to a solitary HM site. Though the A_2_B unit carries MTase domains in both A subunits, it still needs extra A subunits (or excess A_2_B protein) to transfer that single methyl group to the substrate (38). Strikingly, the relatively slow reactions at reduced TstI concentrations revealed an initial lag phase in the progress of the reaction, after which subsequent transfers occurred at an accelerated rate (Supplementary Figure S4): the lag phase was over too quickly to detect in the faster reactions at higher TstI concentrations.

The 50*S^M^* and the 50*S_M_* oligoduplexes (Figure 1) carry HM TstI sites with 6mA in place of the target adenine in top or bottom strands respectively. When incubated with the TstI protein and ^3^H-labelled SAM, the extent of labelling of the HM duplexes proceeded to half the level observed with the UM 50*S* duplex as these can accept only one methyl group, to whichever strand is unmethylated. No significant difference was observed between the rates of transfer to the top or bottom strands (on 50*S_M_* or 50*S^M^* respectively) but in both cases the rates were about 20 times faster than that on the UM duplex (Figure 8b). Hence, even though the TstI RM protein can methylate UM sites as fast as it can cleave them, it operates much more efficiently, albeit solely as a MTase, at HM sites. This feature concurs with the primary role of a MTase from a RM system, which is to convert HM DNA left after semi-conservative replication to the FM state before the next round of replication (6,7,10). The faster rate of transfer to a HM site also accounts for why a lag phase precedes the methylation reaction on an UM duplex (Supplementary Figure S4): the latter most likely involves a slow initial transfer of one methyl group to one strand to yield a HM intermediate which is then methylated rapidly in the other strand (16).

The MTases from many Type II RM systems act equally at UM and HM sites (15,16) but those from Type I systems often show either no or substantially reduced activities at UM relative to HM sites: those in the Type IA subset such as EcoKI and EcoBI have no activity at UM sites (47,48) while Type IB and IC MTases such as EcoAI and EcoR124I are typically ∼20-fold less active at UM sites (49,50). The MTase from the TstI protein resembles more closely a Type IB or IC MTase than a MTase from its own Type II classification, while BcgI behaves like a Type IA MTase.

## CONCLUSIONS

The BcgI and the TstI REases both fall in the IIB subset of Type II systems as both make two DSBs at their sites, one either side of the recognition sequence, and excise from the rest of the DNA a small fragment carrying the site (2,17). Yet these two RM proteins have remarkably few similarities to each other in terms of either subunit organisation or mode of action. The differences may well originate from the fact that the A_2_B protomer of BcgI possesses per DNA recognition unit two active sites for its nuclease and two for its MTase, while each subunit of TstI has a DNA recognition domain coupled to only one catalytic unit for each activity. The difficulties inherent in the latter stoichiometry have been noted before (10). The principal similarity is that they both need to interact with two copies of their respective recognition sequences for their REase reactions but they act as MTases at solitary sites. Yet even though their REase reactions both involve two sites, they process the DNA to different extents: primarily two DSBs by TstI, one at each site; four by BcgI, two at each site. In addition, while both BcgI and TstI carry out their MTase reactions at lone sites, they do so with different protein assemblies.

**Figure 9.**
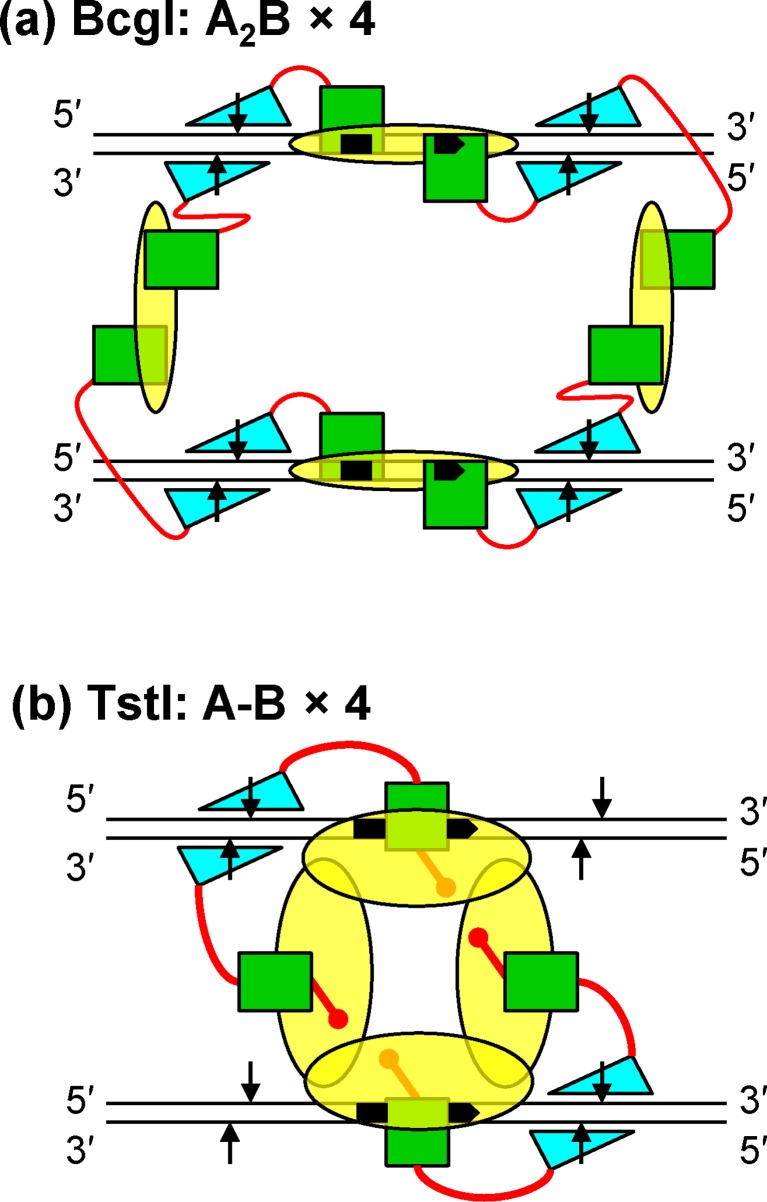
Domain organisation in DNA cleavage complexes for BcgI (**a**) and TstI (**b**). In both schematics, two DNA segments are represented as sets of parallel black lines, with 5′-3′ polarities as indicated. The bipartite recognition sites are filled in black, with arrowheads to note their orientations. The scissile phosphodiester bonds are marked with vertical arrows. In both proteins, DNA recognition domains are shown as yellow ovals, methyltransferase domains as green squares and endonuclease domains as cyan triangles (the latter oriented to match strand polarity): covalent inter-domain linkers are drawn in red. In BcgI (a), methyltransferase and endonuclease domains are in one polypeptide (A) and the DNA recognition domain in another (B). DNA cleavage by BcgI is thought to involve an assembly of four A_2_B protomers and two copies of the recognition sequence, so that all eight scissile bonds are engaged by one of the eight endonuclease domains in the (A_2_B)_4_ assembly [from (22)]. In TstI (b), each of the four polypeptides in the tetramer carries endonuclease, methyltransferase and DNA recognition domains, covalently linked in a single polypeptide in a 1:1:1 stoichiometry. The tetramer bound to two DNA sites thus has only four endonuclease domains and in the scheme shown in (b), two of these are placed to make a DSB upstream of the upper site and the other two a DSB downstream of the lower site. Transfer of the endonuclease domains to the intact loci would almost certainly require the dissociation of the partially cleaved DNA followed by its re-association in converse configurations.

The synaptic complex formed by the BcgI protein across two copies of its cognate sequence cleaves the DNA at all eight scissile bonds in a concerted process, converting the DNA directly to the final products with concomitant release of the excised fragments (37). Concurrent action at eight bonds presumably needs eight active sites for phosphodiester hydrolysis. Hence, in its REase role, BcgI probably forms an assembly containing eight A subunits, most likely an (A_2_B)_4_ tetramer spanning the two sites (Figure 9a).

On the other hand, TstI exists as a tetramer in solution (Figure 2), as seen before with the related protein AloI (25). The catalytic turnover of TstI in both its REase and MTase reactions at sub-stoichiometric protein concentrations (Figure 3, Supplementary Figure S4) excludes the involvement of higher-order assemblies containing two or more tetramers. The tetramer has the potential to bind four cognate sites at the same time. [TstI–DNA complexes failed to enter polyacrylamide gels, thwarting attempts to observe discrete complexes with one, two or more duplexes (data not shown).] But the addition of the specific 50*S* duplex failed to enhance its cutting of the two-site plasmid (Supplementary Figure S2d) whereas it had enhanced the cutting of the one-site plasmid (Figure 4b, Supplementary Figure S2a). Hence, it is unlikely that TstI can act concurrently at more than two sites. But with only four active sites, it is impossible for TstI to work in the same way as BcgI and cut concertedly all eight bonds within a single synaptic complex (Figure 9b). Instead, the TstI REase cuts either just one bond at a time or makes just one DSB at each copy of its recognition sequence, depending on the stability of the synaptic complex (Figure 4). At 37°C, the complex formed *in cis*, on a DNA with two TstI sites, had a sufficiently long lifetime to allow the enzyme to make a DSB at both sites, but when raised to its physiological temperature only one site was cut before the complex collapsed (Figure 4d). The primary reaction of the TstI REase is thus the introduction of one DSB at each site (Figure 4c), much like a tetrameric Type IIS enzyme, except that in the case of TstI, the DSB can be either upstream or downstream of the site. However, having deployed its four active sites to make one DSB at both sites (Figure 9b), the TstI enzyme will almost certainly have to dissociate from the DNA before re-orienting itself to cut on the other side of each site. The MspJI endonuclease provides a precedence for this scheme as it is a tetramer that can bind four DNA segments but which focuses its active sites onto two of the bound segments (42). But while MspJI uses four active sites to cut four phosphodiester bonds, two at each copy of its recognition site, the TstI tetramer has to cut eventually eight bonds.

The MTase function of TstI can act at solitary sites as a single tetramer, transferring to the DNA one methyl group at a time (Figure 7). The lag in the rate of methylation of an UM site shows that the two strands are methylated in sequential steps (Supplementary Figure S4). The TstI MTase acts much more rapidly at HM than at UM sites (Figure 8), yet its rate at UM sites is still comparable to the rate at which the protein makes its first cuts at an UM site. Hence, in reactions containing both REase and MTase co-factors, Mg^2+^ and SAM respectively, TstI fails to cleave all of the DNA due to a fraction becoming modified and thus no longer susceptible to cleavage (Figure 6). Indeed, in reactions containing SAM and Ca^2+^, to allow for methyl transfer but not cleavage, the rate of methyl transfer to UM sites was faster than that measured for the release of the 32-mer in cleavage reactions containing Mg^2+^ (Figures 5 and 8). In the presence of both SAM and Mg^2+^ ions, as must be the case *in vivo*, an UM site is likely to become methylated, and thus protected, before being cut on both sides. The hallmark of a Type IIB system is that the REase cleaves both sides of the site but in the case of TstI, and probably all of the other single-polypeptide Type IIB systems, the excision of the site from the remainder of the DNA is unlikely to ever happen apart from reactions *in vitro* lacking SAM.

## SUPPLEMENTARY DATA

Supplementary Data are available at NAR Online, including Supplementary Methods, Supplementary References, Supplementary Figures S1–S4.

SUPPLEMENTARY DATA
