# Supplementary material for: TstI, a Type II restriction–modification protein with DNA recognition, cleavage and methylation functions in a single polypeptide
Source: Nucleic Acids Res. 2014 Mar 14;42(9):5809–22. doi: 10.1093/nar/gku187 (PMC4027205; doi:10.1093/nar/gku187)
Supplement: SUPPLEMENTARY DATA [file supp_42_9_5809__index.html]

TstI, a Type II restriction–modification protein with DNA recognition, cleavage and methylation functions in a single polypeptide — SUPPLEMENTARY DATA 

# TstI, a Type II restriction–modification protein with DNA recognition, cleavage and methylation functions in a single polypeptide

## SUPPLEMENTARY DATA

**Files in this Data Supplement:**

- SUPPLEMENTARY DATA
