# Supplementary material for: TstI, a Type II restriction–modification protein with DNA recognition, cleavage and methylation functions in a single polypeptide
Source: Nucleic Acids Res. 2014 Mar 14;42(9):5809–22. doi: 10.1093/nar/gku187 (PMC4027205; doi:10.1093/nar/gku187)
Supplement: SUPPLEMENTARY DATA [file supp_gku187_nar-00130-h-2014-File006.pdf]

SUPPLEMENTARY DATA for:

TstI, a Type II restriction-modification protein with DNA recognition, cleavage and methylation functions in a single polypeptide

Rachel M. Smith, Christian Pernstich and Stephen E. Halford*

The DNA-proteins Interaction Unit, School of Biochemistry, University of Bristol, University Walk, Bristol, BS8 1TD, UK

* To whom correspondence should be addressed. Tel: +44 117 3312156; FAX: +44 117 3312168; Email: s.halford@bristol.ac.uk

---

1. Supplementary Methods

Protein Purification

Single colonies of the transformants of *E. coli* T7 Express *lysY/I^q* with either pET15b-TstI or pET21a-TstI were initially grown overnight in 50 ml LB with ampicillin (50 µg/ml) at 37°C. The cultures were then used to inoculate 2 L LB with ampicillin at 25°C and grown to an OD_{600} of ~0.5 before adding IPTG to 0.4 mM. After 18 h, the cells were harvested by centrifugation and stored at -20°C. For the His-tagged protein, the cells carrying pET15b-TstI were suspended in 100 ml Buffer A (20 mM Tris-HCl, pH 8.0, 500 mM KCl, and 10% (v/v) glycerol) supplemented with 20 mM imidazole and two protease inhibitor tablets (Roche). The suspension was fed through a Constant Systems Ltd cell disrupter, briefly sonicated to reduce the viscosity of the solution and the debris removed by centrifugation. All subsequent chromatography steps employed an Äkta Purifier 9000 FPLC system (GE Healthcare, who also supplied all columns noted below): column fractions were examined by SDS-PAGE and, when appropriate, assays for TstI REase activity. The supernatant was applied to a His-trap FF column and the column first washed in Buffer A supplemented with 10 mM imidazole before elution of the bound proteins with a linear gradient from 10 – 300 mM imidazole in Buffer A. Peak fractions were diluted 7-fold with Buffer B (20 mM Tris-HCl, pH 8.0, 1 mM EDTA, 10% (v/v) glycerol) and loaded onto a heparin column. The protein was then eluted from the heparin column with a linear gradient from 50 – 600 mM KCl in buffer B. Finally, it was subjected to gel filtration chromatography (HiLoad Superdex200 16/60) in Buffer B supplemented with 100 mM KCl. Peak fractions were pooled and stored at -80°C. Immediately prior to each experiment, the TstI RM protein was diluted to the requisite concentration in buffer B containing 100 mM KCl.

For the native form of TstI, the T7 Express[pET21a-TstI] cells were suspended in 100 ml buffer B supplemented with 50 mM KCl and two protease inhibitor tablets (Roche) and disrupted as above. The resultant supernatant was subjected to chromatography on heparin-Sepharose followed by Mono-S and then Mono-Q. All three columns were developed with linear gradients from 0.05 to 1 M KCl in buffer B. The fractions from the Mono-Q column that contained TstI were combined and applied to a gel-filtration column equilibrated in buffer B with 100 mM KCl. Active TstI fractions, as judged by endonuclease assays, were pooled, concentrated and stored at -80°C.

Construction of plasmid substrates

The 3806 bp sequence of a plasmid used previously as a substrate for various REases (restriction enzymes), pDG5 (1), lacks the recognition sequence for TstI. The plasmid was cleaved with AatII and ligated to the following oligoduplex,

5′-P-AGGATCCAGCTGTCTGAGATACACTCGT**CAC**GAACAA**TCC**ATCCAGTCTCTGACTATGTACGT-3′
3′-TGCATCCTAGGTCGACAGACTCTATGTGAGCA**GTG**CTTGTT**AGG**TAGGTCAGAGACTGATACA-P-5′

The duplex carries the recognition sequence for TstI (underlined) so this insertion creates a novel 3869 bp plasmid with one TstI site. The novel plasmid was in turn cleaved with ClaI and ligated to a further duplex

5′-P-CGAAGGATCCAGCTGTCTGAGATACACTCGT**CAC**GAACAA**TCC**ATCCAGTCTCTGACTATGT-3′
3′-TTCCTAGGTCGACAGACTCTATGTGAGCA**GTG**CTTGTT**AGG**TAGGTCAGAGACTGATACAGC-P-5′

that also carried a TstI site, to create a 3931 bp plasmid with two TstI sites in inverted orientation 1035 bp apart. The two duplexes noted above carry their TstI sites embedded in 59 bp of identical sequence so that the cognate sites possess identical spacer and flanking sequences including the cleavage loci 7 and 12 bp distant from the site in 5′ and 3′ directions respectively (Figure 1 in main text).

The one-site construct was however cleaved by TstI not only at the newly-introduced recognition site but also, albeit less efficiently, at one additional site (data not shown). Cleavage at this extra site was detected even with TstI protein that had been purified to homogeneity but, apart from the cognate and this one additional site, the DNA was not cut at any other site. A variety of buffer conditions were tested with varied salt concentrations and with the addition of organic solvents, but the ratio of the additional to the anticipated products remained the same. Hence, TstI nuclease reactions at this extra site appears to be distinct from the “star” activity commonly seen with REases: “star” activity is generally enhanced by special buffer conditions such as low salt or the presence of glycerol (2-4) and almost always leads to cleavage of plasmid or phage DNA at multiple locations. Instead, the additional site for TstI appears to be more akin to a secondary site, of the sort seen first with SgrAI (5). In the presence of either its cognate site or the two termini generated by cutting its cognate site, SgrAI can cleave DNA at a discrete set of sites 1 bp different from its 8 bp recognition sequence (6,7).

The approximate location of the additional site was determined by restriction mapping and found to be ~250 bp distant from the newly-introduced cognate site, in the β-lactamase gene present in pDG5. Inspection of the sequence in the vicinity of the mapped location revealed three loci where the sequence differed from the cognate site for TstI by 1 bp:

5′-**CAC**CCAGCG**TtC**-3′
5′-**CgC**CCTTAT**TCC**-3′
5′-**CAC**TCGTTG**TaC**-3′

[Nucleotides in bold denote the 5/6 identity with the specified segments of the cognate sequence for TstI (**CAC**nnnnnn**TCC**) and that in red in lower case marks the single bp deviation.] Site-directed mutagenesis (using the QuikChange XL site-directed mutagenesis kit from Agilent Technologies with the appropriate primers) was applied to change individually one further bp in each of these sequences, to give products that now deviated from the cognate sequence at two positions. In all three cases, the C at the 5′-end of the left-hand segment was changed to a different base but one that retained the amino acid sequence of the β-lactamase protein. C → G mutations at the first and at the second of the sequences noted above had no effect on the additional cleavage but a C → T change at the third site abolished the additional cleavage by TstI.

The sequence 5′-**CAC**TCGTTG**TaC**-3′ at this location thus appears to constitute a secondary site for TstI, but it should be noted that this same sequence appears elsewhere in the derivatives of pDG5 employed here but at these other locations it fails to elicit secondary cleavage activity (data not shown). Secondary site activity by TstI thus seems to involve factors beyond the 12 bp sequence, but which have yet to be established. Nevertheless, this secondary site was removed by site-directed mutagenesis as above from not only the one-site plasmid but also both the two-site plasmid and pDG5 itself. The derivatives of pDG5 with the extra mutation at the secondary site were named as follows: pTST0, the product from pDG5 that now lacks both cognate and secondary sites; pTST1 and pTST2, the constructs noted above, with one cognate and with two cognate sites respectively, but both now lacking the secondary site. All three plasmids were sequenced in their entirety (Eurofins MWG operon): no unanticipated variations were found.

2. Supplementary references

1. Marshall,J.J.T., Gowers,D.M. and Halford,S.E. (2007) Restriction endonucleases that bridge and excise two recognition sites from DNA. *J. Mol. Biol.*, **367**, 419-431.
2. Polisky,B., Greene,P., Garfin,D.E., McCarthy,B.J., Goodman,H.M. and Boyer,H.W. (1975) Specificity of substrate recognition by the *Eco*RI restriction endonuclease. *Proc. Natl. Acad. Sci. U.S.A.*, **72**, 3310-3314.
3. Tikchonenko,T.I., Karamov,E.V., Zavizion,B.A. and Naroditsky,B.S. (1978) *Eco*RI* activity: Enzyme modification or activation of accompanying endonuclease? *Gene* **4**, 195-212.
4. Halford,S.E., Lovelady,B.A. and McCallum,S.A. (1986) Relaxed specificity of the *EcoRV* restriction endonuclease. *Gene*, **41**, 173-181.
5. Bitinaite,J. and Schildkraut,I. (2002) Self-generated DNA termini relax the specificity of *Sgr*AI restriction endonuclease. *Proc. Natl. Acad. Sci. U.S.A.*, **99**, 1164-1169.
6. Hingorani-Varma,K. and Bitinaite,J. (2003) Kinetic analysis of the coordinated interaction of *Sgr*AI restriction endonuclease with different DNA targets. *J. Biol. Chem.*, **278**, 40392-40399.
7. Wood,K.M., Daniels,L.E. and Halford,S.E. (2005). Long-range communications between DNA sites by the dimeric restriction endonuclease SgrAI. *J. Mol. Biol.*, **350**, 240-253.
8. Jurenaite-Urbanaviciene,S., Serksnaite,J., Kriukiene,E., Giedriene,J., Venclovas,C. and Lubys,A. (2007) Generation of DNA cleavage specificities of type II restriction endonucleases by reassortment of target recognition domains. *Proc. Natl. Acad. Sci. U. S. A.*, **104**, 10358-10363.

3. Supplementary Figures

Supplementary Figures S1 – S4 are shown on the following pages.

Smith, Pernstich and Halford

Supplementary Figure 1 (page 1 of 2)

	M I T R D N L K D L L L Q L G F E Q K G
1.	CCATGGGCCATATGATTACGCGTGATAATCTGAAAGATCTGCTGCTGCAGCTGGGTTTTGAACAGAAAG
	N V F S K E F P A I D A Y L R V D F H K E K L
70.	GTAATGTGTTCAGCAAAGAATTTCCGGCAATTGATGCATATCTGCGCGTGGATTTTCACAAAGAGAAAC
	I Y P E D K G F K I N E Q Q I C N F K A K E N
139.	TGATTTATCCTGAAGATAAAGGCTTCAAAATCAACGAGCAGCAAATCTGCAACTTCAAAGCCAAAGAAA
	F V V F E C V H R L F E K G Y K P Q H I E L E
208.	ACTTTGTGGTGTTTGAATGCGTTCACCGCCTGTTTGAAAAAGGTTATAAACCGCAGCATATTGAACTGG
	P R W Q V G H G A S G G R A D I L I R D N D G
277.	AACCGCGTTGGCAGGTTGGTCATGGTGCAAGCGGTGGTCGTGCAGATATTCTGATTCGTGATAATGATG
	K P L L I I E C K T W G D E F N N A W K D T L
346.	GTAAACCGCTGCTGATCATTGAATGTAAAACCTGGGGTGATGAATTTAACAACGCATGGAAAGATACCC
	N G K G Q L F T Y A H Q E R S T Q Y L C L Y A
415.	TGAATGGTAAAGGTCAGCTGTTTACCTATGCACATCAAGAACGTAGCACCCAGTATCTGTGTCTGTATG
	S G L E D G V L K Y V S H I I A V R D N D D I
484.	CCAGCGGTCTGGAAGATGGTGTTCTGAAATATGTGAGCCATATTATTGCCGTGCGTGATAACGATGATA
	L K R K A A E N P P T Y R E A D S A K G L Y E
553.	TCCTGAAACGTAAAGCAGCAGAAAATCCGCCTACCTATCGTGAAGCAGATAGCGCAAAAGGTCTGTATG
	V W K R T Y D A D Y A T R G I F E P D V Q P Y
622.	AAGTTTGGAAACGTACCTATGATGCAGATTATGCAACCCGGGGCATTTTTGAACCGGATGTTCAGCCGT
	Q I G K A K Y T V A D L Q T V T E A D I K P K
691.	ATCAGATTGGTAAAGCAAAATATACCGTTGCCGATCTGCAGACCGTTACCGAAGCCGATATTAAACCGA
	Y H E F A T I L R Q H N V S G R E N A F D K L
760.	AATATCATGAATTTGCCACCATTCTGCGTCAGCATAATGTTAGCGGTCGTGAAAATGCCTTTGATAAAC
	V N L F L C K I V D E K H N P N D L Q F Y W R
829.	TGGTGAACCTGTTCCTGTGTAAAATTGTCGACGAAAAACATAACCCGAACGACCTGCAGTTTTATTGGC
	G I A F D T Y F D L I D R L Q R L Y R D G M R
898.	GTGGTATTGCATTTGATACCTACTTCGATCTGATTGATCGTCTGCAGCGTCTGTATCGTGATGGTATGC
	E F L K E D V T Y I D D R T I R D A F K F F K
967.	GTGAATTCCTGAAAGAAGATGTGACCTATATTGATGATCGCACCATTCGTGATGCCTTCAAATTCTTTA
	N D P D A T R E K I L D Y F R R Q K Y F T N N
1036.	AAAACGATCCGGATGCAACGCGTGAAAAAATCCTGGATTATTTCCGTCGCCAGAAATACTTTACCAACA
	D F S F L D V H N E Q L F Y Q N A A I L L K I
1105.	ACGATTTTAGCTTCCTGGATGTGCACAATGAGCAGCTGTTTTATCAGAATGCAGCAATCCTGCTGAAAA
	V R M L Q D I R L N G D Q Q N Q F L G D M F E
1174.	TCGTACGGATGCTGCAGGATATTCGTCTGAATGGCGATCAGCAGAACCAGTTTCTGGGTGACATGTTCG
	Y F L D Q G F K Q T E G Q F F T P L P V T R F
1243.	AATATTTTCTGGATCAGGGCTTTAAACAGACCGAAGGTCAGTTTTTTACACCGCTGCCGGTTACCCGTT
	I I L S L P L E T I F S D E Q N P P K V I D Y
1312.	TTATCATTCTGAGCCTGCCGCTGGAAACCATTTTTAGTGATGAACAGAATCCTCCGAAAGTGATTGATT
	A C G A G H F L T E M A S Q L R R L R P G Q D
1381.	ATGCATGTGGTGCAGGTCATTTTCTGACCGAAATGGCAAGCCAGCTGCGTCGTCTGCGTCCGGGTCAGG
	V K F Y S K F Y S E F Y G V E K E Y R L S K V
1450.	ATGTGAAATTTTACAGCAAATTCTACAGCGAGTTCTATGGCGTGGAAAAAGAATATCGTCTGAGCAAAG
	A K V S A F M Y N Q D E I N I I Y A D A L V R
1519.	TTGCAAAAGTGAGCGCCTTTATGTATAATCAGGATGAGATCAACATCATCTATGCAGATGCACTGGTTC
	H P D I P E G A F A L L V S N P P Y S V K G F
1588.	GTCATCCGGATATTCCGGAAGGTGCATTTGCACTGCTGGTTAGCAATCCGCCTTATAGCGTTAAAGGTT
	L E T L P K T E R E R Y E L I K T V D P K S Y
1657.	TTCTGGAAACCCTGCCGAAAACCGAACGTGAACGTTATGAACTGATTAAAACCGTTGATCCGAAAAGCT
	P T N D A I E A F F L E R A R Q L L A P H G L
1726.	ATCCGACCAATGATGCAATTGAAGCGTTTTTCCTGGAACGTGCCCGTCAGCTGCTGGCACCGCATGGTC
	A A I I V P S S L L S N T D N I Y T R T R E I
1795.	TGGCAGCAATTATTGTTCCGAGCAGCCTGCTGAGCAATACCGATAACATTTATACCCGTACCCGTGAAA
	L L R N F D I V A L V E L G N G T F G K T G T
1864.	TTCTGCTGCGCAATTTTGATATTGTTGCCCTGGTTGAACTGGGTAATGGCACCTTTGGTAAAACCGGCA
	N T V T L F L R R K D D N P A P A D H Y R N R
1933.	CCAATACCGTTACCCTGTTTCTGCGTCGTAAAGATGATAATCCGGCACCGGCAGATCATTATCGTAATC
	V N A W F N G N M D K D G V F A D A S L L Q A
2002.	GTGTTAATGCATGGTTCAACGGCAACATGGATAAAGATGGCGTTTTTGCAGATGCGAGCCTGCTGCAGG
	Y C D Y I G I P L D D Y R T L L Q S N P S P A
2071.	CGTATTGTGATTATATTGGTATTCCGCTGGATGATTATCGTACACTGCTGCAGAGCAATCCGAGTCCTG
	L L D T E I F K E Y R R A F D S S S E V E K L
2140.	CCCTGCTGGATACCGAAATTTTCAAAGAATACCGTCGTGCGTTTGATAGCAGCAGCGAAGTTGAAAAAC
	K K R K S F Q K K S E D E R Q A E L N R R F I
2209.	TGAAAAAACGCAAATCCTTTCAGAAAAAAAGCGAAGATGAACGTCAGGCAGAACTGAATCGTCGCTTTA

```
         Q  F  A  R  E  I  E  R  E  K  L  Y  Y  F  I  L  A  Y  T  Q  S  C  P
2278.  TTCAGTTTGCACGTGAAATCGAACGCGAGAAACTGTATTATTTCATTCTGGCATATACCCAGAGCTGTC
         V  I  V  V  R  S  P  Q  N  T  E  E  R  K  K  F  L  G  Y  E  W  S  A
2347.  CGGTTATTGTTGTTCGTAGTCCGCAGAATACCGAAGAACGCAAAAAATTCCTGGGCTATGAATGGTCAG
         A  K  G  Q  E  G  I  K  Y  L  H  G  S  I  D  C  I  E  T  P  L  F  D
2416.  CAGCCAAAGGTCAAGAAGGCATTAAATACCTGCATGGTAGCATTGATTGTATCGAAACTCCGCTGTTTG
         P  Q  K  P  D  N  P  E  K  I  S  T  L  I  R  R  N  F  E  G  Q  A  V
2485.  ATCCGCAGAAACCTGATAACCCGGAAAAAATCAGCACCCTGATTCGTCGTAATTTTGAAGGTCAGGCCG
         S  I  P  E  S  L  Q  P  Y  A  T  L  T  R  L  V  D  L  L  D  F  S  R
2554.  TTAGCATTCCGGAAAGCCTGCAGCCGTATGCCACCCTGACCCGTCTGGTTGATCTGCTGGATTTTAGCC
         V  S  F  D  K  Q  I  S  L  A  P  K  K  G  A  T  Q  V  Q  S  K  W  E
2623.  GTGTTAGCTTCGATAAACAAATTAGCCTGGCACCGAAAAAAGGTGCAACCCAGGTTCAGAGCAAATGGG
         V  R  K  V  G  D  V  C  N  F  E  Y  G  K  G  L  P  Q  N  K  R  Q  P
2692.  AAGTTCGTAAAGTTGGTGATGTGTGCAATTTCGAATATGGCAAAGGTCTGCCGCAGAACAAACGTCAGC
         G  P  Y  P  V  I  G  S  N  G  R  V  G  F  H  N  Q  Y  L  V  E  G  P
2761.  CTGGTCCGTATCCGGTGATTGGTAGCAATGGTCGTGTTGGTTTTCATAATCAGTATCTGGTTGAAGGTC
         A  I  I  V  G  R  K  G  T  A  G  A  V  Y  W  E  D  N  N  C  W  P  I
2830.  CGGCTATTATTGTGGGTCGTAAAGGTACAGCCGGTGCAGTGTATTGGGAAGATAATAACTGTTGGCCGA
         D  T  T  F  Y  V  K  L  K  A  S  D  I  S  L  R  Y  L  Y  L  M  L  Q
2899.  TTGATACCACCTTCTATGTTAAACTGAAAGCCAGCGATATTAGCCTGCGTTATCTGTACCTGATGCTGC
         E  L  H  L  D  K  L  S  G  G  V  G  V  P  G  L  N  R  D  D  V  Y  Q
2968.  AAGAACTGCATCTGGATAAACTGAGCGGTGGTGTTGGTGTTCCGGGTCTGAACCGTGATGATGTTTATC
         Q  K  I  P  V  P  P  L  D  V  Q  A  Q  I  V  D  E  C  Q  A  I  D  A
3037.  AACAGAAAATTCCGGTTCCGCCTCTGGATGTCCAGGCACAGATTGTTGATGAATGCCAGGCCATTGATG
         E  V  E  Q  A  E  K  E  V  S  D  C  Y  Q  I  A  K  E  K  V  Q  A  C
3106.  CAGAAGTTGAACAGGCAGAAAAAGAAGTTAGCGATTGTTATCAGATCGCGAAAGAAAAAGTTCAGGCAT
         F  A  Q  G  Q  V  T  A  L  G  T  L  V  H  I  N  R  E  S  T  D  P  T
3175.  GTTTTGCACAGGGTCAGGTTACCGCACTGGGCACCCTGGTTCATATTAATCGTGAAAGCACCGATCCGA
         Q  F  S  E  K  S  F  I  Y  V  D  I  G  N  V  E  K  G  T  G  V  I  D
3244.  CCCAGTTTAGCGAAAAAAGTTTTATCTATGTGGATATTGGCAACGTCGAAAAAGGCACCGGTGTTATTG
         Y  S  Q  V  I  T  G  K  D  A  P  S  R  A  R  R  I  A  P  K  G  S  V
3313.  ATTATTCACAGGTTATTACCGGCAAAGATGCACCGAGCCGTGCACGTCGTATTGCACCGAAAGGTAGCG
         I  I  S  T  V  R  P  N  L  R  G  F  A  F  I  D  R  D  T  A  D  C  V
3382.  TGATTATTAGCACCGTGAGGCCTAATCTGCGTGGTTTTGCCTTTATTGATCGTGATACCGCAGATTGTG
         F  S  T  G  F  A  V  L  E  S  K  D  E  S  V  L  K  N  K  S  L  F  Y
3451.  TTTTTAGCACCGGTTTTGCAGTTCTGGAATCAAAAGATGAAAGCGTGCTGAAAAACAAAAGCCTGTTTT
         A  F  M  F  S  D  D  L  M  A  Q  M  I  D  A  M  G  K  A  A  Y  P  S
3520.  ATGCCTTCATGTTCTCCGATGATCTGATGGCACAGATGATCGATGCAATGGGTAAAGCCGCATATCCGA
         I  N  Q  T  D  I  E  N  L  R  I  R  V  P  D  V  Q  A  Q  E  K  L  I
3589.  GCATTAATCAGACCGATATTGAAAATCTGCGTATTCGTGTGCCGGATGTGCAGGCCCAAGAAAAACTGA
         Q  E  L  D  K  L  E  T  Q  L  Q  S  A  R  A  V  I  A  S  A  P  A  R
3658.  TCCAAGAACTGGACAAACTGGAAACACAGCTGCAAAGCGCACGTGCAGTTATTGCAAGCGCACCGGCAC
         R  R  A  V  L  K  K  Y  L  E  N  A  *
3727.  GTCGTCGTGCGGTTCTGAAAAAATACCTCGAGAATGCATAAGGATCCAAGCTT
```

Figure S1. Reconfigured gene sequence. The sequence shown is the 5′-3′ strand of the duplex that was synthesised in order to generate the TstI RM protein. The segment in light blue encodes the same amino acid sequence (indicated in single-letter code) as the *tsti* gene from *Thermus scotoductus* RFL1 (GI:149391961, AM410095.1) but it differs from the genomic sequence as a result of codon optimisation for expression in *Escherichia coli* and the introduction of unique restriction sites at the start (NcoI, NdeI), at the end (BamHI, HindIII) and at various points within the gene (in 5′-3′ order: MluI, SmaI, EcoRI, SalI, BsiWI, PciI, StuI and XhoI). External sites are highlighted in yellow and internal in dark blue. The new internal sites were mostly placed at or near the boundaries proposed for the REase, MTase and DNA recognition domains of the multifunctional polypeptide (8), to permit future manipulations of individual domains of the protein.

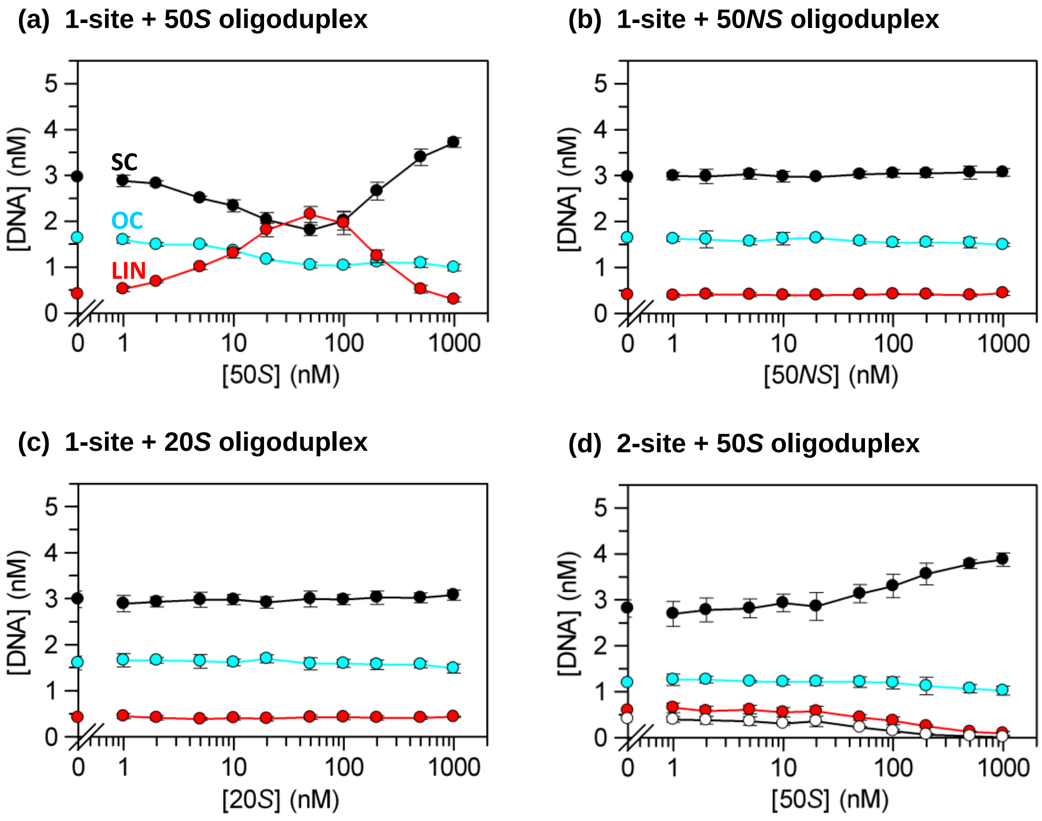

Figure S2: Activation of plasmid cleavage by oligoduplexes. Reactions in buffer R at 37°C contained 5 nM plasmid DNA (^{3}H-labelled), 12.5 nM TstI tetramer and the indicated concentration of the designated oligoduplex: (**a**) pTST1 with 50*S*; (**b**) pTST1 with 50*NS;* (**c**) pTST1 with 20*S;* (**d**) pTST2 with 50*S*. Reactions on the one-site (pTST1) and two-site (pTST2) plasmids were stopped after 5 and 0.5 min respectively. For each set, a control reaction without oligoduplex was carried out. The reaction products were analysed as in Figure 3 (main text) and the concentration of each form of the plasmid plotted as a function of the oligoduplex concentration: SC, black circles; OC, cyan circles; LIN, red circles; and, in (**d**) only, L1 + L2 , unfilled circles. The logarithmic x-axes are broken so that the concentrations of each form present in the incubation without oligoduplex can also be shown.

(a) Reaction scheme

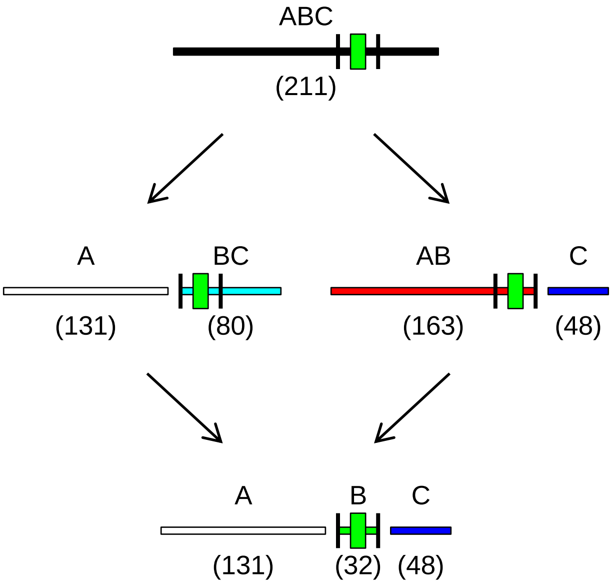

(b) Result

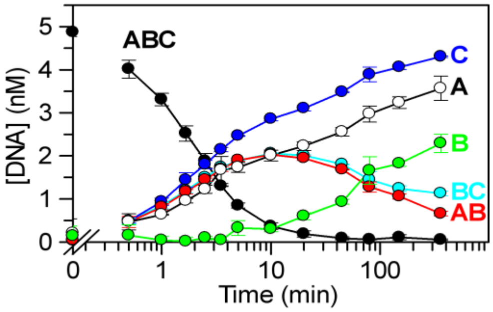

Figure S3. (**a**) The substrate named ABC was used to monitor the complete profile of a TstI reaction on a DNA with one recognition site for TstI (green box, cleavage loci on either side marked by vertical bars). It was produced by PCR amplification of a section of pTST1 spanning its TstI site. Also shown are the products from cutting the DNA at the left-hand locus (A and BC, unfilled and cyan boxes respectively), at the right-hand locus (AB and C, in red and blue respectively), and at both loci (A and C as above, B [the 32-mer] in green). The number below each DNA denotes its size in bp. (**b**) The reaction contained 5 nM ABC and 12.5 nM TstI protein in Buffer R at 37°C. Samples were removed from the reactions at the indicated times (the break in the logarithmic time scale is to allow for the inclusion of the zero time point), quenched and then analysed by electrophoresis through a 15% polyacrylamide gel in TBE in order to separate on the basis of their distinct sizes all six DNA species (the ABC substrate and all five products: A, BC, AB, C and B). The gel was stained with SYBR Safe and fluorescence recorded by PhosphorImager as in Materials and Methods. Concentrations of all six species were evaluated from the records with ImageQuant software, and are shown in the same colours as in (a). All data points are the means from three repeats, with error bars for standard deviations.

Smith, Pernstich and Halford
Supplementary Figure 4

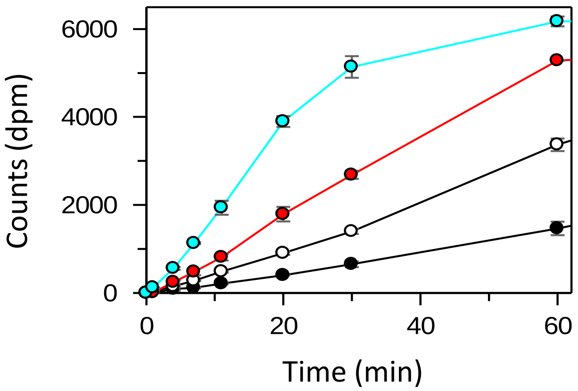

Figure S4. Methylation of UM sites at varied protein concentrations. Reactions, in buffer M at 37°C, contained 100 nM 50*S* and one of the concentrations of TstI protein listed below. Samples were taken from the reaction mix at the indicated times and then analysed as in Materials and Methods to determine the level of radiolabel transferred from ^{3}H-SAM into the DNA: each dpm value shown is the average from three independent experiments with error bars to indicate standard errors of the mean. The concentrations of TstI tetramer were: 15 nM, in black; 30 nM, unfilled circles; 50 nM, in red; 100 nM, in cyan.
